# Effectiveness of brief interventions and contacts after suicide attempt: a systematic review and meta-analysis

**DOI:** 10.1016/j.eclinm.2026.103824

**Published:** 2026-03-12

**Authors:** Stephanie Homan, Marta Anna Marciniak, Sofia Michel, Anna-Marie Bertram, Charlotta Rühlmann, Annamária Pethő, Lara Kirchhofer, Leonie Biele, Robin Segerer, Philipp Homan, Sebastian Olbrich, Rory C. O'Connor, Birgit Kleim

**Affiliations:** a Department of Adult Psychiatry and Psychotherapy, Psychiatric University Clinic Zurich and University of Zurich, Zurich, Switzerland; b Experimental Psychopathology and Psychotherapy, Department of Psychology, University of Zurich, Zurich, Switzerland; c Healthy Longevity Center, University of Zurich, Zurich, Switzerland; d Department of Clinical Psychology and Psychotherapy, University of Bern, Bern, Switzerland; e Department of Psychology, University of Basel, Basel, Switzerland; f Department of Psychology, Education and Child Studies, Erasmus University Rotterdam, Rotterdam, the Netherlands; g Department of Clinical Psychology, Semmelweis University, Budapest, Hungary; h Department of Psychology, Developmental and Personality Psychology, University of Basel, Basel, Switzerland; i Neuroscience Center Zurich, University of Zurich and Swiss Federal Institute of Technology Zurich, Zurich, Switzerland; j Suicidal Behaviour Research Laboratory, School of Health & Wellbeing, University of Glasgow, Glasgow, UK

**Keywords:** Brief interventions and contacts, Psychosocial intervention, Suicide attempt, Randomized controlled trials, Meta-analysis

## Abstract

**Background:**

Following a suicide attempt, only a third of people receive outpatient treatment, highlighting the need for low-threshold brief interventions and contacts (BICs). We aimed to examine the effectiveness of BICs.

**Methods:**

In this systematic review and meta-analysis, we searched MEDLINE, Embase, Cochrane CENTRAL, PsycInfo, Web of Science, and ProQuest, and gray literature from inception to June 18, 2025, for randomized controlled trials of BIC in adults following a suicide attempt. Studies were included if they investigated the effectiveness of BICs compared with control for adults (aged 18–65) after a suicide attempt, intended to reduce suicide re-attempts, suicidal ideation, self-harm, non-suicidal self-injury, or linkage to mental health (MH) services. The main outcomes included suicide re-attempts, self-harm, suicidal ideation, non-suicidal self-injury (NSSI), and linkage to MH services. We extracted raw frequency counts and means/SDs for use in random-effects meta-analyses of odds ratios and standardized mean differences, respectively. Quality of the evidence was assessed with RoB 2 and GRADE. This study is registered with PROSPERO, CRD42022271143.

**Findings:**

Thirty-six trials (9552 participants; 1993–2025) were included, with 33 trials eligible for meta-analysis. Suicide re-attempts were significantly reduced after BICs compared with control (moderate-certainty evidence; OR = 0.72, 95% CI [0.54, 0.95]; I^2^ = 56.8%; n = 23 studies). Reductions in suicidal ideations were observed after BICs compared with control (low-certainty evidence; SMD = −0.20, 95% CI [−0.36, −0.05]; I^2^ = 63.4%; n = 15 studies). We found no evidence for reductions in self-harm recurrence (very low-certainty evidence; OR = 0.66, 95% CI [0.22, 1.97]; I^2^ = 80.9%; n = 4 studies) and increases in linkage to MH services (very low-certainty evidence; OR = 2.25, 95% CI [0.71, 7.17]; I^2^ = 89.8%; n = 6 studies). There were too few studies for an investigation of NSSI. Most trials showed some concerns (22 studies, 61%), while fewer showed high risk of bias (7 studies, 19%). Risk of bias, heterogeneity, and imprecision contributed to the downgrading of certainty.

**Interpretation:**

Our findings have important implications for clinical practice and suicide prevention. Even when delivered in a single session, BICs can effectively reduce the recurrence of suicide attempts and may also decrease suicidal thoughts. Although conclusions for self-harm recurrence and linkage to MH services were limited, these interventions can be considered a practical and potentially essential element of suicide prevention strategies. Further high-quality trials are needed to confirm effects across additional outcomes and populations.

**Funding:**

The study was supported by funding from the Swiss National Science Foundation (501100001711-205913); the 10.13039/501100008464EMDO Foundation of the University of Zurich; the HOLCIM enterprise for the Promotion of Scientific Further Education.

**Research in context**

**Evidence before this study**

Suicide attempts are a major public health concern, and brief interventions and follow-up contacts have shown promise in reducing risk and supporting care. However, guidelines are limited, and it remains unclear which components are most effective and which patients benefit most. We searched five databases (MEDLINE, Embase, Cochrane Database of Systematic Reviews, APA PsycInfo, and Web of Science) up to June 18, 2025, using terms including ‘suicide attempts’, ‘suicidal ideation’, ‘self-harm’, ‘brief interventions’, and ‘systematic reviews’ to identify systematic reviews and meta-analyses of RCTs evaluating brief interventions and contacts for adults after suicide attempts. Prior evidence suggested that these interventions can reduce suicide re-attempts, but results varied by intervention type, outcomes, follow-up duration, and study quality. To address these limitations, we conducted a systematic review and meta-analysis, examining both suicide-specific outcomes and intervention types.

**Added value of this study**

To our knowledge, this is the first systematic review and meta-analysis to evaluate brief interventions and follow-up contacts for adults after suicide attempts across multiple suicide-specific outcomes, including re-attempts, suicidal ideation, self-harm, non-suicidal self-injury, and linkage to mental health services. Synthesizing 33 RCTs, we found a modest but significant reduction in suicide re-attempts, and smaller but meaningful reductions in suicidal ideation, with variable effects on self-harm and other outcomes. Subgroup and meta-regression analyses showed that interventions including follow-up contacts, psychoeducation, and structured psychosocial components were generally most effective, with efficacy varying by intervention type, follow-up duration, and patient population. These findings provide a framework to guide secondary prevention strategies and clinical decision-making.

**Implications of all the available evidence**

Our findings suggest that brief interventions and follow-up contacts can meaningfully reduce suicide re-attempts, supporting their role as scalable components of post-attempt care and informing policy and clinical decision-making. There is also an indication of benefits for suicidal ideation, though evidence is less consistent. Effects on self-harm and non-suicidal self-injury remain unclear. Future well-powered trials with standardized outcomes and comparative designs are needed to clarify which intervention types work best, for whom, and under what circumstances, guiding effective implementation within broader suicide prevention strategies.

## Introduction

For suicide prevention efforts to be more effective, the World Health Organization (WHO) called for prioritizing increased access to and quality of mental healthcare.^1^ Improving access to care is vital for two reasons. First, most people who die by suicide have had recent contact with healthcare services, 80% within a year and 44% within a month of their death.^2^ These encounters present a critical opportunity to detect suicidal thoughts and behaviors (STBs) and initiate support. Second, treatment engagement is often poor, with only 35% attending a follow-up appointment within a week of hospital discharge, and 55% within a month.^3^^,^^4^ Given the elevated risk of re-attempts among people with prior suicide attempts,^5^ interventions must be feasible and supportive of ongoing care coordination. However, traditional treatments often involve long wait times^6^ and multiple sessions, which may not align with the immediate needs of people after a suicide attempt.

Pioneering work^7^ showed that simple follow-up letters significantly reduced suicide deaths. Since then, a wide range of brief interventions has emerged, among which the Safety Planning Intervention (SPI),^8^^,^^9^ has received particular attention. The SPI, along with related formats such as crisis response plans and coping cards, involves a structured, collaborative process in which personal warning signs, coping strategies, and sources of support are identified and developed to prevent suicidal crises.^9^ These safety-planning type interventions are recommended as best practice by major guidelines such as NICE in the UK and the Suicide Prevention Resource Center in the USA, and have been specifically evaluated in a meta-analysis by Nuij and colleagues,^9^ which examined the efficacy of SPI on suicidal thoughts and behaviors. Beyond safety planning, brief interventions also include brief psychotherapy, phone calls, postcards, or crisis cards.^10^ Overall, these approaches provide structured, time-limited support to reduce suicide risk and promote follow-up care, particularly in the critical time after a hospital-treated suicide attempt. Delivered by clinicians or trained paraprofessionals, they typically involve 1 to 12 sessions and are offered across a wide range of treatment and emergency settings.^11^ Although brief interventions are considered effective and more accessible and cost-effective,^12^ their therapeutic mechanisms remain unclear but may include enhanced social support and suicide prevention literacy,^13^ safety planning, psychoeducation,^14^^,^^15^ therapeutic engagement, long-term follow-up,^15^ and follow-up time not exceeding six months.^16^ Consequently, some reviews show promising effects,^9^^,^^14^, ^15^, ^16^, ^17^, ^18^, ^19^, ^20^, ^21^, ^22^, ^23^, ^24^ while others show no effect,^25^, ^26^, ^27^ or mixed results.^28^^,^^29^ Moreover, implementing brief interventions and contacts (BICs) consistently across diverse clinical settings remains challenging, as maintaining fidelity to intervention protocols can be difficult due to variations in staff training, resources, and organizational contexts, potentially affecting outcomes and limiting scalability. These uncertainties highlight a critical gap in the evidence: Which intervention components work best, and for whom?

The primary aim of this meta-analysis was to systematically examine the evidence on brief interventions and contacts (BICs) for adults following a suicide attempt, providing an update to earlier reviews on this topic (e.g.,^25^), which suggested beneficial effects on suicidal thoughts and behaviors (STB). BICs may influence a broad spectrum of outcomes, including suicide attempts, suicidal ideation, self-harm/NSSI, and service engagement; examining this spectrum allows a more comprehensive understanding of where these interventions are effective. Moreover, in recent years, novel approaches and delivery methods have emerged, including digital and blended formats, that were not covered in earlier syntheses. Our secondary aims were to investigate the moderating effects of intervention type and population on outcomes, thereby advancing our understanding of potential mechanisms of action and informing the development of more targeted interventions.

## Methods

We followed the PRISMA-P guidelines^30^ and adhered to the recommendations outlined in the Cochrane Handbook for Systematic Reviews of Interventions.^31^

### Search strategy and selection criteria

In this systematic review and meta-analysis, we included all randomized controlled trials (RCTs) that evaluated specific brief psychosocial interventions in adults (18–65 years) who sought treatment following a suicide attempt. “Suicide attempt” was defined as a “self-inflicted, potentially injurious behavior with a nonfatal outcome, for which there is evidence (either explicit or implicit) of intent to die.”^32^ Studies including participants with suicidal ideation only were excluded. BICs were defined following Stanley et al.^11^ as structured, short-term approaches to reduce suicide risk, with 1–4 sessions (ultra-brief) or 6–12 sessions (brief) that are implemented in diverse treatment and emergency care settings and compared with nonsuicide-specific treatment as usual. Interventions could be delivered in person or online, and involve psychotherapy or brief contacts (e.g., calls, postcards). Although this definition covers conceptually diverse modalities, we retained it to align with the established literature and to ensure comparability with prior meta-analyses. We grouped interventions based on previously suggested categories^29^: (1) Brief interventions (i.e., psychotherapy-based approaches), (2) remote contact interventions (e.g., letters, phone calls, postcards), (3) multimodal interventions (i.e., combinations of brief interventions and contact-based elements), and (4) ‘other’ (i.e., all other forms such as case management).

We searched MEDLINE (Ovid), Embase, Cochrane CENTRAL, PsycInfo, Web of Science, and ProQuest (including dissertations and conference proceedings) from inception to June 18, 2025, using a strategy developed with an information specialist (RS) and structured according to the PICO framework. The population block included adults with a documented suicide attempt or self-harm; the intervention block captured brief psychosocial and contact-based approaches such as safety planning, brief cognitive–behavioral therapy, problem-solving therapy, caring contacts, postcards, and follow-up calls; and the outcome block covered suicide re-attempts, suicidal ideation, non-suicidal self-injury, self-harm, and linkage to mental health services. A validated RCT filter combining publication type and keyword terms was applied.^9^, ^10^, ^11^^,^^14^^,^^17^^,^^19^^,^^27^^,^^29^ No restrictions were placed on language, publication year, or country. The complete MEDLINE Ovid search history is provided in Supplementary Figure S1, with corresponding strategies adapted for the other databases.

Title-abstract and full-text screening were conducted independently by SM and LB on Rayyan, a web-based platform.^33^ Screenings were compared by SH, and discrepancies were resolved through discussion. During the title-/abstract screening, we excluded 68 studies, primarily because they included participants without a history of suicide attempt (see Supplementary Table S1). For more details on the methods, see the Supplementary Material.

### Data analysis

Data from the included studies were independently extracted by AP and AMB. We collected information on general study characteristics, including first author, title, year, design, country, study aim, and key conclusions; participant details, such as population description, method of suicide attempt, clinical setting, inclusion criteria, diagnoses, assessment tools, numbers randomized and analyzed, age, sex and gender distribution; intervention and comparator characteristics, including type, format, number of sessions, and follow-up details (period and frequency); and outcomes, including primary and secondary endpoints, raw data, and timing of assessments. For the primary endpoint, we prioritized the time point specified for the primary outcome; if this was not provided, we extracted data from the time point closest to intervention completion, consistent with our focus on post-intervention effectiveness. When information was missing or incomplete, study authors were contacted. SH reviewed all extracted data, and any discrepancies were resolved through discussion. We examined five primary outcomes: (1) suicide re-attempts, (2) suicidal ideation, (3) NSSI, (4) self-harm, and (5) linkage to mental health (MH) services. Linkage to MH services was defined as engagement with an MH care provider *versus* re-hospitalization.^14^^,^^29^

For the categorical outcomes (i.e., ‘re-attempts’, ‘NSSI’, and ‘linkage to MH services’), we extracted raw event numbers (frequency counts). For the continuous outcomes (i.e., ‘suicidal ideation’ and ‘self-harm’), we had anticipated extracting raw scale scores (with *M* and *SD*) as reported by previous reviews (e.g.,^29^). However, while suicidal ideation was reported in this format, self-harm was presented as a frequency statistic and was therefore extracted as raw event counts. For the analysis, we calculated pooled odds ratios (ORs) for the categorical outcomes and standardized mean differences (SMDs) for the continuous outcomes.^34^

For studies with multiple treatment arms^35^, ^36^, ^37^ that compared multiple interventions to the control group, we used the recommended approach^38^ and combined data from similar intervention arms. For studies with the Zelen design,^39^^,^^40^ we extracted data from all randomized participants to preserve the integrity of randomization.^41^ For studies with adjusted effect sizes, we extracted the observed, unadjusted effect sizes to avoid inconsistent estimates. Data on participants’ sex were extracted when reported. Due to inconsistent reporting and the lack of sex-disaggregated outcomes across studies, sex- or gender-specific effects could not be examined quantitatively.

To evaluate potential bias, SH and MM independently assessed all included studies using the Cochrane Risk of Bias tool (RoB 2).^42^ Discrepancies were resolved through discussion. Inter-rater reliability was moderate (overall weighted *k* = 0.59, *p* < 0.001) with an overall agreement of 77% (Supplementary Figures S2 and S3). The certainty of evidence for each outcome was assessed using the Grading of Recommendations Assessment, Development and Evaluation (GRADE) approach.^43^ Two reviewers (SH and MM) independently performed the GRADE assessments, and disagreements were resolved through discussion with a third reviewer (LB).

For the meta-analysis, we included 33 data sets, each contributing one to three outcomes. We investigated (1) post-treatment effects and (2) the effect of time (i.e., follow-up period) on the outcomes.

Post-treatment effects were examined using random-effects meta-analyses. For categorical outcomes, we pooled log Ors, which were back-transformed to OR for reporting. For continuous outcomes, SMDs were pooled directly. To investigate how treatment effects evolved over time, we used a multivariate random-effects meta-analytic approach including all available follow-up assessments from each study. This approach accounts for the dependency among multiple effect sizes within studies and models follow-up time continuously, allowing a more precise and nuanced assessment of temporal trends in treatment efficacy across varying follow-up intervals.

As measures of heterogeneity, we used the *Q*-test^44^ and the *I*^2^ statistic.^34^ While the *Q*-test provides a measure of whether the observed heterogeneity in effect sizes is greater than expected by chance, the *I*^2^ indicates the proportion of variance not attributable to sampling error.^45^^,^^46^ To assess small-study effects, including publication bias, we used Peter's regression test (categorical outcomes) and Egger's test (continuous outcomes). To identify outliers, we used studentized residuals and Cook's distances.^47^ Additionally, we examined funnel plots for result inconsistencies. Finally, to assess the robustness of the meta-analytic findings, we conducted leave-one-out sensitivity analyses, iteratively omitting each study and examining the impact on the pooled effect sizes.

Subgroup analyses were conducted post-hoc to explore whether intervention effects varied by intervention type (brief interventions, remote contact interventions, multimodal interventions, or other) and to provide descriptive insights into heterogeneity observed across studies. Subgroup meta-analyses were performed separately for each intervention type where sufficient data were available.

We also conducted post-hoc meta-regression analysis using mixed-effects models to examine potential moderators of treatment effects, including intervention type (e.g., brief interventions, remote contact interventions, multimodal interventions, or others), studied population (e.g., college students, ED patients, general population, inpatient soldiers, inpatients, outpatients), intervention format (i.e., ultra-brief [1–4 sessions] vs. brief [6–12 sessions]), and year of publication. For year of publication, we tested both a simple model with year as the only predictor and a more complex model including population and intervention type as covariates; models were compared using ANOVA. Statistical significance of the meta-regression coefficients was evaluated using z-tests and 95% confidence intervals (CI). All meta-regressions were considered exploratory due to the limited number of studies and uneven distribution of moderators across studies.

This paper was written in Rmarkdown using RStudio (version 1.4.1106)^48^ and the packages: rmarkdown (version r 2.30),^49^ knitr (version r 1.51),^50^ papaja (version r 0.1.4),^51^ and robvis (version r 0.3.0).^52^ Meta-analyses were conducted using metafor (version r 4.8.0).^47^ The code is freely available online to ensure transparency and reproducibility of the analyses (https://osf.io/brgm7/).

This review was pre-registered with PROSPERO (CRD42022271143). One deviation from the protocol occurred, i.e., we conducted meta-regressions to examine intervention type and population.

### Role of the funding source

The funders of the study had no role in study design, data collection, data analysis, data interpretation, or writing of the report. All authors had full access to all the data in the study. SH and BK had final responsibility for the decision to submit the manuscript for publication.

## Results

**Fig. 1:**
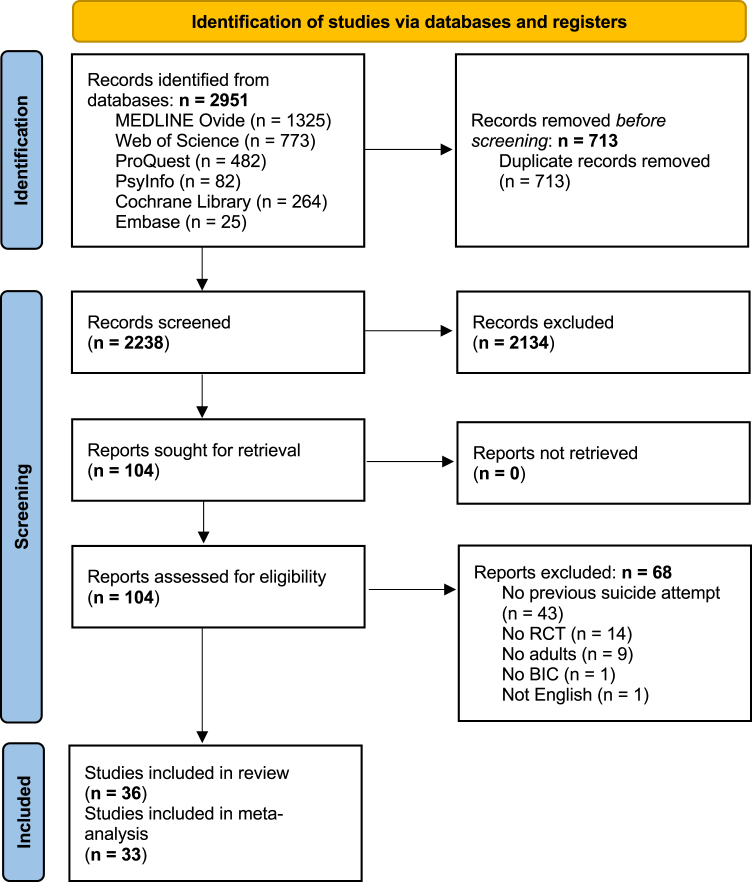
**PRISMA Flow Diagram.** Initially, 2951 records were identified through database searches. Following the removal of duplicates, 2238 records remained for screening, resulting in the exclusion of an additional 2134 records. A total of 104 full-text articles were assessed for eligibility, of which 68 did not meet the inclusion criteria. Finally, a total of 36 were included in the review. Because 3 studies reported on none of the outcomes of interest, only 33 were included in the meta-analysis.

Of the 2951 initially identified studies, 36 met the inclusion criteria and were accessible. As 3 of those reported on none of the outcomes, only 33 studies were included in the meta-analysis (Fig. 1). The studies were published between 1993 and 2025 and conducted throughout the world: 15 in Europe,^37^^,^^39^^,^^40^^,^^53^, ^54^, ^55^, ^56^, ^57^, ^58^, ^59^, ^60^, ^61^, ^62^, ^63^, ^64^ nine in America,^65^, ^66^, ^67^, ^68^, ^69^, ^70^, ^71^, ^72^, ^73^ six in Asia,^35^^,^^74^, ^75^, ^76^, ^77^, ^78^ four in Middle Eastern countries,^79^, ^80^, ^81^, ^82^ and one in Australia and New Zealand.^36^ One study was conducted in multiple countries.^83^

Sample sizes at baseline varied across studies, with 12–945 participants per intervention group. Overall attrition rates ranged from 0 to 52, with a mean of 17.99 (*SD* = 14.76). Attrition tended to increase with longer follow-up periods (Supplementary Figure S4). Attrition rates were generally comparable across intervention and control groups. Across studies, the mean number of participants lost was 0.48 higher in the intervention group than in the control group (paired t-test: *t* (32) = 0.13, *p* = 0.90, 95% CI [−7.31, 8.27]).

Of the 9213 participants investigated, over half were female (5364, 58.20%; Table 1) with an average age of 35.68 years (*SD* = 8.12). The studied population was predominantly clinical (86.10%, 31/36 studies) and primarily recruited from the ED. All participants had experienced at least one suicide attempt. The 13 studies that reported on the suicide method identified self-poisoning as the most frequent method (61.50%, 8/13 studies).^54^^,^^57^^,^^63^^,^^64^^,^^66^^,^^79^^,^^81^^,^^83^

**Table 1:** Descriptives of the studies’ population.

Studies	Population	Brief interventions and contacts				Control condition			
		N	Female	Age (M)	Age (SD)	N	Female	Age (M)	Age (SD)
Armitage et al., 2016	Inpatients	77	NA	31.57	16.07	73	NA	28.25	11.44
Arvilommi et al., 2022	ED patients	119	NA	30.92	3.76	120	NA	28.25	11.44
Cedereke et al., 2002	Inpatients	107	62	32.20	13.30	109	52	32.00	12.40
Chen et al., 2013	Outpatients	373	71	40.00	18.00	388	72	42.00	18.00
Conner et al., 2021	Inpatients	16	243	39.80	14.00	18	275	40.00	16.00
Diefenbach et al., 2024	Inpatients	103	12	42.80	15.20	106	10	38.40	17.80
Fleischmann et al., 2008	ED patients	922	58	33.10	12.40	945	59	32.50	12.80
Ghahramanlou-Holloway et al., 2020	IP soldiers	77	518	NA	NA	73	475	NA	NA
Guthrie et al., 2001	ED patients	12	5	30.30	11.40	12	5	27.80	9.30
Gysin-Maillart et al., 2016	ED patients	58	33	NA	NA	61	33	NA	NA
Kapur et al., 2013	ED patients	60	36	36.50	14.30	60	30	39.20	14.60
Kaslow et al., 2010	Inpatients	33	NA	NA	NA	33	NA	NA	NA
Keyworth et al., 2025	General hospital patients	130	121	NA	NA	87	87	NA	NA
LaCroix et al., 2018	IP soldiers	520	347	46.29	14.17	520	351	45.70	14.26
Lin et al., 2019	College students	18	6	28.90	8.60	18	5	33.00	10.80
Lin et al., 2020	ED patients	42	38	20.40	0.76	40	34	20.47	0.71
Malakouti et al., 2021	Inpatients	72	50	29.50	NA	75	56	34.00	NA
Matsubara et al., 2019	ED patients	153	105	NA	NA	152	99	NA	NA
McAuliffe et al., 2014	ED patients	24	NA	NA	NA	24	NA	NA	NA
Monn et al., 2025	Inpatients	222	64	33.40	11.50	211	65	33.60	12.10
Morgan et al., 1993	Inpatients	46	24	34.20	13.50	46	20	34.50	15.00
Mouaffak et al., 2015	ED patients	101	NA	27.40	NA	111	NA	32.50	NA
Mousavi et al., 2016	ED patients	160	113	39.00	13.00	160	111	38.60	13.30
Mousavi et al., 2017	ED patients	29	27	27.07	7.79	26	21	29.69	7.73
O’Connor et al., 2015	Inpatients	30	20	NA	NA	30	22	NA	NA
O’Connor et al., 2020	Inpatients	15	1	43.67	13.13	15	7	39.02	14.43
O’Connor et al., 2022	Inpatients	23	10	43.26	2.48	25	12	41.96	2.70
Owens et al., 2020	ED patients	80	50	36.10	16.10	40	25	37.00	14.10
Rahmani et al., 2025	Inpatients	30	19	34.80	13.90	32	21	35.60	14.00
Rudd et al., 2015	IP soldiers	25	14	20.60	2.60	25	11	21.50	3.20
Sheehan et al., 2023	General population	76	12	27.18	6.25	76	7	27.62	6.19
Stewart et al., 2009 (1st arm)	ED patients	19	7	46.90	13.84	19	8	49.60	13.35
Stewart et al., 2009 (2nd arm)	ED patients	32	NA	NA	NA	35	NA	NA	NA
Vaiva et al., 2006 (1st arm)	ED patients	147	115	38.00	12.00	312	221	35.00	11.00
Vaiva et al., 2006 (2nd arm)	ED patients	147	105	35.00	12.00	312	221	35.00	11.00
Vaiva et al., 2018	Inpatients	493	312	38.40	13.40	494	314	38.10	13.10
Wang et al., 2016	General population	33	24	39.13	10.25	34	23	36.78	11.87
van der Sande et al., 1997	Inpatients	140	92	35.80	15.60	134	88	36.80	14.60

*Note.* ED, emergency department; M, mean; N, sample size; NA, not available; SD, standard deviation.

Most studies (72.20%, 26/36 studies) focused on reducing the recurrence of suicide attempts.^37^^,^^39^^,^^40^^,^^53^^,^^56^, ^57^, ^58^, ^59^, ^60^^,^^63^, ^64^, ^65^, ^66^, ^67^^,^^69^^,^^71^^,^^72^^,^^74^, ^75^, ^76^, ^77^, ^78^, ^79^, ^80^, ^81^ Half of the studies (50%, 18/36 studies) investigated suicidal ideation.^35^^,^^36^^,^^39^^,^^40^^,^^53^^,^^54^^,^^56^^,^^57^^,^^65^, ^66^, ^67^, ^68^, ^69^, ^70^^,^^72^^,^^76^^,^^81^^,^^82^ Self-harm was addressed in 4 studies^54^^,^^55^^,^^57^^,^^62^ that targeted these deliberate self-injury behaviors, often as part of broader suicide prevention efforts. Improving linkage to MH services was the focus of 9 studies^37^^,^^53^^,^^55^^,^^59^^,^^60^^,^^71^^,^^76^^,^^77^^,^^79^ that aimed to enhance motivation for care engagement. NSSI was assessed by only one study,^56^ indicating a gap in the current literature. Three studies reported on none of the primary outcomes, but instead focused on suicide mortality,^83^ psychiatric readmissions,^61^ and depression severity.^73^

A variety of measurement tools and procedures were used to assess the outcomes. Suicide attempts were mostly assessed through self-reports or patient health records (PHR) (68.0%, 17/25 studies). Only in a few studies, clinical scales were used (32.0%, 8/25 studies); these included the Suicidal Behaviors Questionnaire (SBQ),^84^ Columbia-Suicide Severity Rating Scale (C-SSRS),^85^ British Psychiatric Morbidity Survey (BPMS),^86^ CMSADS-L Short form, Chinese version of Pierce Suicide Intent Scale,^87^ and the Suicide Attempt Self-Injury Interview.^88^ Suicidal ideation was mainly assessed quantitatively with clinical scales (94.4%, 17/18 studies), including the SBQ, C-SSRS, Scale of Suicide Ideation,^89^ Adult Suicidal Ideation Questionnaire,^90^ Scale for Suicide Ideation-Worst,^89^ Beck Scale for Suicidal Ideation,^91^ BPMS, and the Suicidal Risk Inventory.^92^ Only one study used self-constructed questions (5.6%, 1/18 studies). Self-harm was assessed through self-report and PHR (100%, 4/4 studies). Non-suicidal self-injury was assessed with a clinical scale in the one study that considered this outcome.^56^ Linkage to MH services was mainly assessed qualitatively through (88.9%, 8/9 studies). Only one study used a clinical scale, the Health Services and Medication Use (11%, 1/9 studies).^93^

Brief psychotherapeutic interventions were investigated by 17 studies.^35^^,^^36^^,^^54^^,^^56^^,^^57^^,^^61^^,^^62^^,^^66^^,^^67^^,^^69^, ^70^, ^71^, ^72^, ^73^^,^^75^^,^^76^^,^^82^ The average number of sessions was 4.97 (SD = 2.72; range: 1–12), with each session lasting approximately 80.07 min. Remote contact interventions were investigated by 11 studies,^37^^,^^53^^,^^55^^,^^59^^,^^60^^,^^63^^,^^74^^,^^77^^,^^78^^,^^80^^,^^81^ involving 3.90 contacts on average (SD = 3.0; range: 1–8). Multimodal interventions were investigated by four studies.^39^^,^^40^^,^^58^^,^^65^ The average number of sessions was 3.25 (SD = 0.5; range: 3–4) lasting approximately 78.75 min, together with six remote contacts on average (SD = 0; range: 6–6). The category ‘other’ included four studies^64^^,^^68^^,^^79^^,^^83^ with 11.50 contacts on average (SD = 3.54; range: 9–14), and included psychoeducation with brief contacts and brief admission.

Only one study^56^ used digital technologies. All studies had at least one follow-up contact with professionals over an extended period (Table 2; follow-up *M* = 1.94, SD = 1.28). Most studies evaluated outcomes at 12 months following the index suicide attempt (see also Supplementary Figure S5).

**Table 2:** Descriptives of the studies’ interventions.

Studies	Name	Groupe	Brief interventions		Brief contacts		Fus (number of FU assessments)	Control	Aim	Effective
			Sessions	Duration	Frequency	Duration				
Armitage et al., 2016 (1st arm)	SGI	BI	2	NA			1	TAU	Test self-generated implementation intention (SGI) to reduce STBs.	yes
Armitage et al., 2016 (2nd arm)	VHS	BI	2	NA			1	TAU	Test volitional help sheet (VHS) to reduce STBs.	yes
Arvilommi et al., 2022	ASSIP + TAU	MI	3	60–90	6	NA	2	active	Assess Attempted Suicide Short Intervention Program (ASSIP) compared to Crisis Counseling to prevent SA.	no
Cedereke et al., 2002	TC	RCI			2	20–45	2	no calls	Investigate the influence of telephone contacts (TC) on SB.	no
Chen et al., 2013	PCI	RCI			1	NA	1	no postcards	Evaluate the effectiveness of crisis postcard intervention (PCI) on SB.	no
Conner et al., 2021	mASSIP	MI	4	60–90			3	active	Examine a modified Attempted Suicide Short Intervention Program (mASSIP) for substance use disorder patients after SA.	NA
Diefenbach et al., 2024	bCBT inpatient + TAU	BI	4	60–90			6	TAU	Determine if brief CBT (bCBT) for inpatients reduces STBs.	yes
Fleischmann et al., 2008	BIC	Other	1	60	9	NA	1	TAU	Compare BIC with TAU on suicide mortality in LMIC.	yes
Ghahramanlou-Holloway et al., 2020	PACT	BI	6	60–90			3	E-UC	Examine Post-Admission Cognitive Therapy (PACT) for SA.	no
Guthrie et al., 2001	PIT	BI	4	50			1	TAU	Compare the impact of a brief psychodynamic interpersonal therapy (PIT) with TAU for SI.	yes
Gysin-Maillart et al., 2016	ASSIP + TAU	MI	3	60–90	6	NA	4	TAU	Compare Attempted Suicide Short Intervention Program (ASSIP) with TAU on reducing SA.	yes
Kapur et al., 2013	Leaflet, TC & letters	RCI			8	NA	1	TAU	Assess the impact of BCI (i.e., leaflet, telephone contact, and letters) to reduce SH.	yes
Kaslow et al., 2010	Nia	Other	10	90			2	TAU	Examined the efficacy of a culturally informed group intervention (Nia) for SI.	yes
Keyworth et al., 2025	eVHS	BI	NA	NA			1	VHS f. phy. activity	To explore whether an online volitional help sheet (eVHS) prevents SH.	no
LaCroix et al., 2018	CT + EUC	BI	6	60–90			3	E-UC	Analyze efficacy of cognitive therapy (CT) among service members with PTSD for SA reduction.	no
Lin et al., 2019	DBT Skills Training Group	BI	8	120			4	active	Dialectic Behavior Therapy (DBT) Skills Training to reduce SA.	no
Lin et al., 2020	bCBT + TAU	BI	6	NA			2	TAU	Examine brief CBT (bCBT) plus standard care for SA.	no
Malakouti et al., 2021	Educational intervention & contacts	Other	1	40–45	14	15–20	2	TAU	Investigate a brief educational intervention and contact program for SB.	yes
Matsubara et al., 2019	BCI + TAU	RCI			3	NA	1	TAU	Determine effectiveness of combined telephone calls and postcard (BCI) on SB.	no
McAuliffe et al., 2014	PS skills training	BI	6	120			2	TAU	Evaluate efficacy of a structured group problem solving (PS) skills training for SH reduction.	no
Monn et al., 2025	ASSIP + TAU	MI	3	90	6	NA	1	TAU	Re-evaluate the efficacy of Attempted Suicide Short Intervention Program (ASSIP) for reducing SA.	no
Morgan et al., 1993	Green Card	RCI			1	NA	1	CAU	Investigate the effect of a Green Card to encourage help seeking behaviour.	no
Mouaffak et al., 2015	OSTA	RCI			4	NA	1	TAU	Test FU-intervention (OSTA) to reduce SA and increase linkage to MH services.	no
Mousavi et al., 2016	TI	RCI			8	20	1	Face-to-face FU	Comparison of telephone delivered intervention (TI) versus face-to-face for SA.	no
Mousavi et al., 2017	TI	RCI			8	20	1	Face-to-face FU	Compared the efficacy of telephone contacts (TC) versus face-to-face follow-up for SI.	yes
O'Connor et al., 2015	TMBI + TAU	BI	1	30–60			1	CAU	Test the feasibility of a Teachable Moment Brief Intervention (TMBI) after SA.	no
O'Connor et al., 2020	TMBI	BI	1	30–60			3	CAU	Evaluate the Teachable Moment Brief Intervention (TMBI) for increasing linkage to MH services.	no
O'Connor et al., 2022	SPI + TC + TAU	BI	2–6	15	5		1	TAU	Test the feasibility of a safety plan (SPI) with telephone follow-ups (TC) for SB.	NA
Owens et al., 2020	PST	BI	6	60			2	TAU	Evaluate problem-solving therapy (PST) to reduce repetition of SH.	yes
Rahmani et al., 2025	ISTDP	BI	5	session 1 & 2 4 h, 3–5 90 min			NA	medication	Evaluate the efficacy of intensive short-term dynamic psychotherapy (ISTDP) for SI.	yes
Rudd et al., 2015	bCBT + TAU	BI	12	60/90			5	TAU	Test brief CBT (bCBT) for the reduction of SB.	yes
Sheehan et al., 2023	2Share	BI	3	120			1	WL	Evaluated a peer-led strategic disclosure intervention (2Share) for SA reduction.	yes
Stewart et al., 2009 (1st arm)	bCBT	BI	4	60			1	TAU	Compare brief CBT (bCBT) to TAU for the treatment of SI.	no
Stewart et al., 2009 (2nd arm)	PST	BI	7	60			1	TAU	Test problem-solving therapy (PST) to reduce SI.	yes
Vaiva et al., 2006 (1st arm)	TC	RCI			1	NA	1	TAU	Test telephone contact (TC) one month after discharge for SA reduction.	yes
Vaiva et al., 2006 (2nd arm)	TC	RCI			1	NA	1	TAU	Test telephone contact (TC) three months after discharge for SA reduction.	no
Vaiva et al., 2018	AlgoS + TAU	RCI			1–5	NA	2	TAU	Evaluate efficacy of an intervention algorithm (AlgoS) for SB.	no
Wang et al., 2016	Coping Cards	RCI			6	NA	1	TAU	Examine the use of coping cards to reduce subsequent SA.	yes
van der Sande et al., 1997	Intensive inpatient & community intervention	Other	4	NA			3	CAU	Assess the effectiveness of an intensive in-patient intervention after SA.	no

*Note.* BIC, Brief intervention and contact; CAU, care as usual; CBT, cognitive behavioral therapy; CC, crisis counseling; E-UC, Enhanced usual care; FU, follow-up; LMIC, low and middle income countries; MH, mental health; MI, multimodal intervention; RCI, remote contact intervention; SA, suicide attempts; SB, suicidal behavior; SH, self-harm; SI, suicidal thoughts; STB, suicidal thoughts and behaviors; TAU, treatment as usual; WL, Waiting list.

Of the 36 included studies, seven studies were assessed with a low risk of bias,^39^^,^^40^^,^^55^^,^^62^^,^^63^^,^^66^^,^^69^ seven were considered high risk,^61^^,^^65^^,^^70^^,^^75^^,^^80^^,^^81^^,^^83^ and 22 were rated as having some concerns.^35^, ^36^, ^37^^,^^53^^,^^54^^,^^56^, ^57^, ^58^, ^59^, ^60^^,^^64^^,^^67^^,^^68^^,^^71^, ^72^, ^73^, ^74^^,^^76^, ^77^, ^78^, ^79^^,^^82^ High-risk assessments were primarily driven by deviations from intended interventions (D2) and bias in the measurement of the outcome (D4) (Supplementary Figures S2 and S3).

**Fig. 2:**
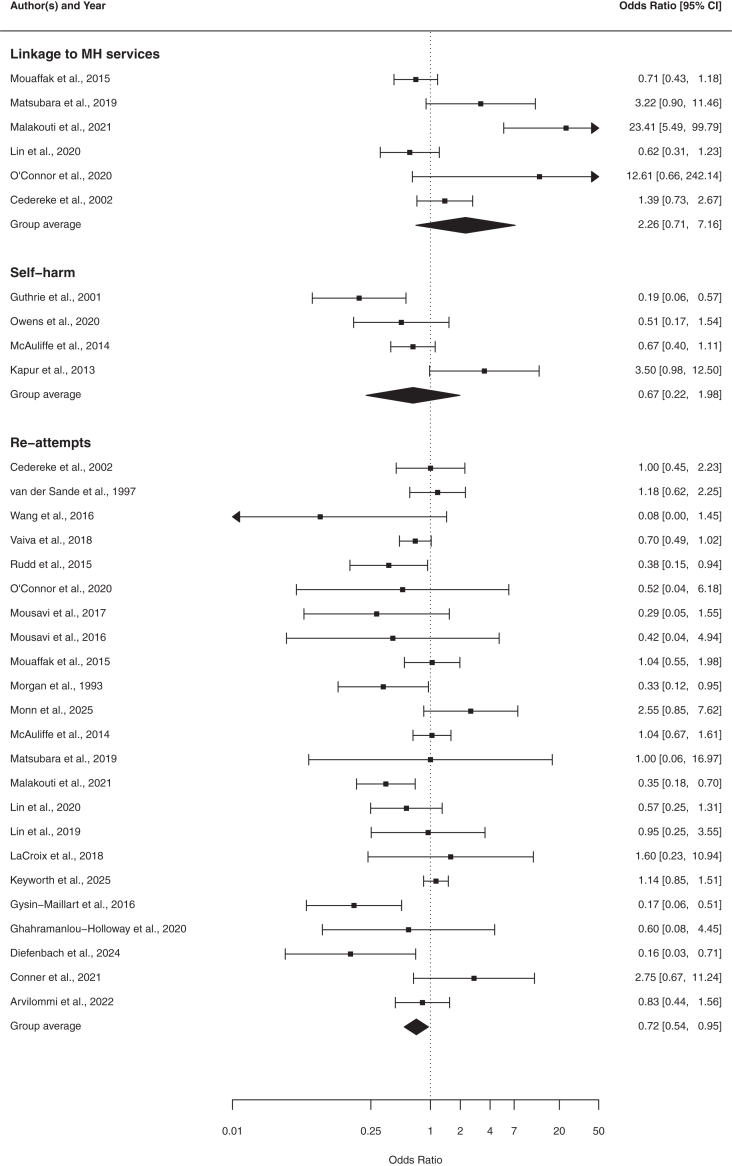
**Meta-Analysis of post-treatment re-attempts, self-harm, and linkage to mental health services: Odds Ratios.** The forest plot presents the odds ratios (OR) for the meta-analyses of categorical outcomes along with the corresponding 95% confidence intervals (CIs) for brief interventions and contacts versus control.

**Fig. 3:**
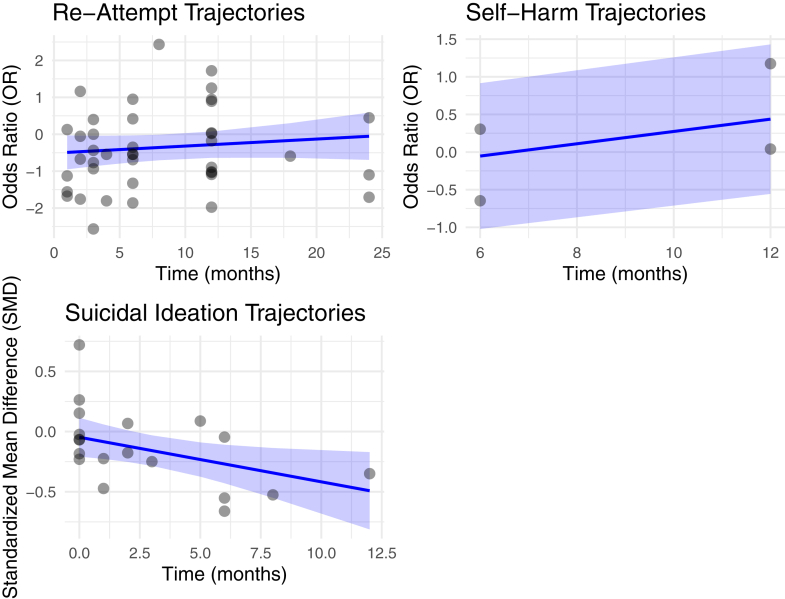
**Symptom trajectories.** The line plot illustrates predicted estimates with 95% confidence intervals alongside observed study-level effect sizes over time (i.e., for all examined follow-up timepoints) for the outcomes for which a multivariate meta-analysis was performed.

We compared the effect of BICs to control at post-treatment and over the follow-up period. BICs had a significant effect in reducing suicide re-attempts post-treatment over an average follow-up period of 10 months (OR = 0.72, 95% CI [0.54, 0.95], *n* = 23; Fig. 2; Supplementary Table S2) compared with control. Heterogeneity was moderate (*Q* (22) = 45.23, *p* = 0.0025; *I*^2^ = 56.8%). No outliers were detected (studentized residual <3.07), and no studies were overly influential according to Cook's distances. Funnel plots did not indicate publication bias (see Supplementary Figure S6), and Peter's regression test was non-significant (*p* = 0.14). The trim-and-fill analysis imputed four right-sided studies and produced an attenuated, non-significant adjusted effect (OR = 0.84, 95% CI [0.61, 1.15]). Given the method's instability under heterogeneity, the pattern of results did not provide strong evidence that publication bias meaningfully influenced the conclusions (Supplementary Figure S7). We investigated the heterogeneity source in a subsequent leave-one-out sensitivity analysis, repeating the meta-analysis iteratively, each time omitting one study. Across all iterations, the pooled effect size remained negative, of similar magnitude (OR range = 0.68–0.78), and significant. Furthermore, we found no effect of time on re-attempts (*β* = 0.02, 95% CI [−0.02, 0.06], *p* = 0.31; Fig. 3; Supplementary Table S3), suggesting that the treatment effect on the odds of re-attempts did not show a systematic change over time. Heterogeneity was substantial (*Q* (40) = 69.76, *p* = 0.0018), which indicated that considerable variability in effect sizes remained unexplained.

**Fig. 4:**
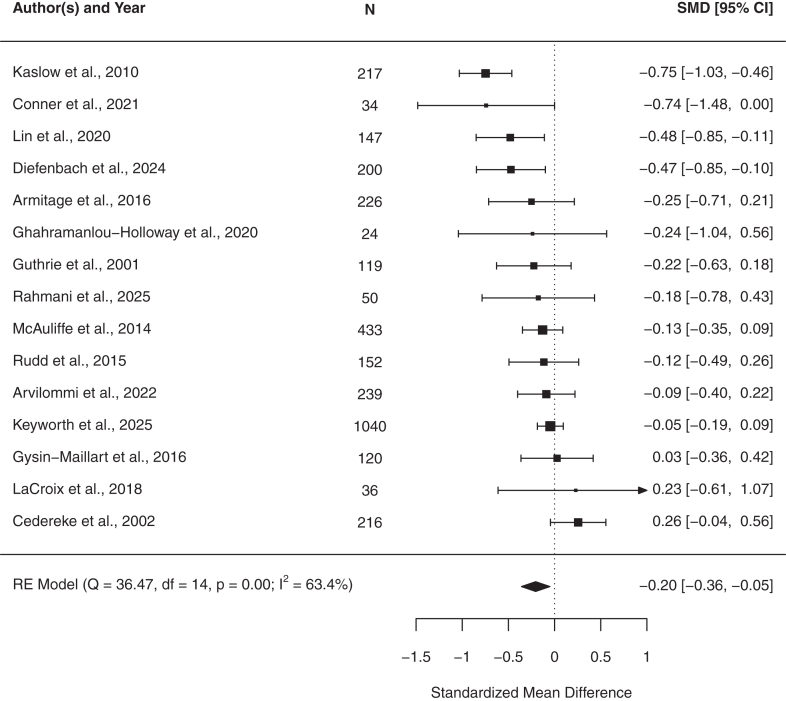
**Meta-analysis results of post-treatment suicidal ideation.** The forest plot presents the meta-analysis of standardized mean differences (SMD) along with the corresponding 95% confidence intervals (CIs) for the continuous outcome comparing brief interventions and contacts to the control group.

Further, BICs were effective in reducing suicidal ideation post-treatment over an average follow-up period of 5 months (SMD = −0.20, 95% CI [−0.36, −0.05], *n* = 15; Fig. 4; Supplementary Table S2) compared with control. Heterogeneity was substantial (*Q* (14) = 36.47, *p* = 0.0009, *I*^2^ = 63.4%). No outliers were detected (max studentized residual <2.94) and no studies were overly influential according to Cook's distances. Funnel plots did not indicate publication bias (see Supplementary Figure S6), and Egger's test was non-significant (p = 0.68). The trim-and-fill procedure did not impute any missing studies, resulting in an adjusted effect identical to the original estimate (SMD = −0.20, 95% CI [−0.36, −0.05]; Supplementary Figure S7). One trial^57^ showed potential influence, but its removal in a sensitivity analysis produced nearly identical results (*Q* (13) = 36.38, *p* = 0.0005; *I*^2^ = 64.10%), indicating robustness. Leave-one-out analysis confirmed the pooled effect remained negative, similar in magnitude (range = −0.24 to −0.14) and statistically significant. Time significantly moderated effects, with a slight decrease over the follow-up period (*β* = −0.04, 95% CI [−0.07, −0.0042]; Fig. 3; Supplementary Table S3), indicating attenuation of treatment effects over time. Heterogeneity was substantial (*Q* (17) = 37.24, *p* = 0.0031), which indicated that considerable variability in effect sizes remained unexplained.

We found no evidence that BICs were effective in reducing self-harm post-treatment over an average follow-up period of 10 months (OR = 0.66, 95% CI [0.22, 1.97], *n* = 4; Fig. 2; Supplementary Table S2) compared with control. Heterogeneity was substantial (*Q* (3) = 11.79, *p* = 0.0081; *I*^2^ = 80.9%). No outliers were detected (max studentized residual <2.94), and no studies were overly influential according to Cook's distances. Funnel plots did not indicate publication bias (see Supplementary Figure S6), and Peter's regression test was non-significant (p = 0.73). The trim-and-fill procedure did not impute any missing studies, resulting in an adjusted effect identical to the original estimate (OR = 0.66, 95% CI [0.22, 1.97]; Supplementary Figure S7). With only 4 studies, these estimates should be interpreted cautiously as the low statistical power limits their informativeness, and results should be regarded as exploratory rather than conclusive. Additionally, we found no moderating effect of time (*β* = 0.08, 95% CI [−0.15, 0.31], *p* = 0.49; Fig. 3; Supplementary Table S3), suggesting that the treatment effect on the odds of self-harm did not show a systematic change over time. Heterogeneity was low and not significant (*Q* (2) = 5.51, *p* = 0.063).

We found no evidence that BICs improved post-treatment linkage to MH services over an average follow-up period of 11 months (OR = 2.25, 95% CI [0.71, 7.17], *n* = 6; Fig. 2) compared with control. Heterogeneity was high (*Q* (5) = 28.51, *p* < 0.0001, *I*^2^ = 89.8%). An examination of the studentized residuals identified one study^56^ with a value slightly above ±2.64, suggesting it may be a potential outlier. According to Cook's distances, no studies were considered overly influential. The funnel plot was too sparsely populated to meaningfully judge asymmetry (see Supplementary Figure S6), and Peter's regression test was significant (p = 0.0013). A trim-and-fill analysis imputed two studies on the left side of the funnel, yielding a substantially attenuated adjusted effect (OR = 1.09, 95% CI [−0.26, 4.61]) relative to the original estimate (Supplementary Figure S7). Given the very high heterogeneity (I^2^ = 93.69%) and the small number of studies, these results should be interpreted cautiously and do not materially change the conclusion that there is no reliable evidence for an effect of BICs on linkage to MH services. Leave-one-out sensitivity analysis showed that no single study substantially changed the overall effect. The effects of time could not be examined, as only two studies were available for this analysis.

We conducted subgroup analyses to explore whether the effects of interventions varied by intervention type, given the heterogeneity observed across studies and the limitations of meta-regression in detecting differences with sparse data. For suicide attempts, brief interventions (k = 9) showed a non-significant trend toward reduced risk (OR = 0.75, 95% CI [0.49, 1.14], p = 0.17) with moderate heterogeneity (I^2^ = 48%), whereas multimodal interventions (k = 4) showed no effect (OR = 0.96, 95% CI [0.28, 3.32], p = 0.95) with substantial heterogeneity (I^2^ = 83%), reflecting considerable uncertainty (Supplementary Table S4a). No eligible studies were available for remote contact interventions. For suicidal ideation, sufficient data were available only for brief interventions (k = 10), which demonstrated a small but statistically significant reduction (SMD = −0.18, 95% CI [−0.30, −0.05], p < 0.01) with low heterogeneity (I^2^ = 22%; Supplementary Table S4b). These findings indicate that effects were most consistent for brief interventions, whereas evidence for other intervention types was sparse or highly heterogeneous. Taken together, these subgroup analyses provide descriptive insights into potential differences across intervention types, which we further examined using exploratory meta-regression analyses.

Exploratory mixed-effects meta-regression analyses were conducted to examine whether selected study-level characteristics explained between-study heterogeneity. Moderators included intervention type, population, intervention format, study risk of bias, and year of publication. Across outcomes, none of these moderators were significantly associated with effect sizes when considered individually (all p-values >0.05; see Supplementary Tables S5–S14). For year of publication specifically, no significant time trends were observed for suicide attempts (β = 0.0043, p = 0.80), suicidal ideation (β = −0.01, p = 0.58), or linkage (β = 0.07, p = 0.46). Exploratory models including intervention type and population as covariates explained most between-study heterogeneity (R^2^ = 95%), with some suggestion that “Other” interventions were associated with larger effects on suicidal ideation (β = −0.41, p = 0.078). These findings suggest that variation in outcomes is primarily attributable to differences in intervention type and target population rather than publication year. Given the limited number of studies and uneven distribution across categories, all meta-regression results are considered exploratory and should be interpreted with caution. We conducted sensitivity analyses to assess the impact of studies with a high risk of bias on the estimate of treatment effectiveness. The results showed that repeating the meta-analyses for these outcomes did not alter the direction of the overall effect size or its significance (‘re-attempts’: original: −0.33, reduced: −0.34; ‘suicidal ideation’: original: −0.20, reduced: −0.16; see Supplementary Table S15).

Overall certainty of evidence ranged from low to very low across outcomes. Evidence was most frequently downgraded due to imprecision, driven by small numbers of studies and wide confidence intervals that included both benefit and harm (Table 3). Additional downgrading occurred for risk of bias and inconsistency for selected outcomes. Detailed GRADE assessments and effect estimate for all outcomes are presented in the Summary of Findings tables (for a more detailed view, see the GRADE profiles in Supplementary Table S16).

**Table 3:** Summary of findings table.

Explanations. a. Visual inconsistency and statistical analysis also showed heterogeneity.

b. Visual inspection of the forest plot and statistical measures indicated considerable heterogeneity, with very high inconsistency across studies (I2 > 80%), suggesting substantial variability in effect estimates that could not be explained.

c. The pooled effect estimate was based on only a few studies and had a wide confidence interval crossing the line of no effect and including both clinically important benefit and possible harm. The total sample size did not meet the optimal information size, indicating very serious imprecision.

d. The evidence was based on a single study assessed narratively. The absence of a pooled effect estimate and failure to meet optimal information size result in very serious imprecision.

e. Funnel plot asymmetry was detected using regression testing (Egger test p = 0.001), suggesting the presence of small-study effects. The direction of asymmetry indicates that smaller studies may overestimate the effect, raising concerns about possible publication bias. The certainty of evidence was downgraded by one level for publication bias.

## Discussion

In this systematic review and meta-analysis, we synthesized over two decades (1993–2025) of research on BICs from 36 RCTs and 9213 suicide survivors. Our main findings are two-fold: (1) BICs significantly reduced suicide re-attempts compared to control conditions, despite their brevity; (2) they may lead to significant reductions in suicidal thoughts compared to control conditions. While the direction of effects was generally consistent across intervention types, the magnitude of these effects is modest, and confidence intervals, particularly for suicidal ideation, indicate uncertainty. Conceptual heterogeneity and the limited number of studies in certain intervention subgroups warrant cautious interpretation regarding mechanisms and generalizability. These findings contribute to clarifying the potential impact of BICs and highlight priorities for future research to identify which intervention components and delivery modes are most effective for suicide attempts versus suicidal ideation. Notably, there were too few studies that assessed self-harm and NSSI in this context.

BICs effectively reduced suicide re-attempts post-treatment, and this effect remained stable over time, with no evidence of systematic change. This is an important finding given the severity of suicidal crises and the public health implications. These low-intensity interventions are feasible in emergency settings, require minimal training, and can be delivered in a single session.^14^ Their effectiveness aligns with growing support for single-session approaches in MH care.^9^^,^^94^ In contrast, traditional psychotherapy is resource-intensive, less accessible, and often only yields modest effects.^12^ In addition, we also observed reductions in suicidal ideation following BICs post-treatment. However, this effect attenuated over time; with longer follow-up periods, the interventions had weaker effects in reducing suicidal thoughts. This effect may reflect the strength of brief interventions to address both immediate cognitive-emotional states and volitional factors that contribute to suicidal behavior. The *integrated motivational-volitional* (IMV) model^95^ offers a framework to interpret these findings. It posits that suicidal behavior emerges when volitional factors (e.g., impulsivity, access to means, entrapment) facilitate the transition from ideation to action. Brief interventions may disrupt this phase by equipping individuals with strategies to manage crises and inhibit suicidal behavior. In this sense, they function as volitional interventions, an increasingly recognized target for suicide prevention.^96^ Evidence from previous work shows mixed effects of BICs on suicidal ideation, with some studies reporting positive effects^18^^,^^22^^,^^24^ and others finding no significant impact.^9^ Taken together, these findings suggest that while BICs may offer short-term benefits for suicidal ideation, their effects may diminish without ongoing or supplementary intervention.

A key goal of BICs is to strengthen linkage to the healthcare system. Ensuring continued engagement with at-risk individuals and facilitating access to MH professionals may help prevent suicide. Active outreach has already been shown to reduce future suicidal behavior.^97^ Within frameworks such as the IMV model^98^ and the *interpersonal theory of suicide*,^99^ maintaining contact through the healthcare system may alleviate feelings of isolation, burdensomeness, and shame, often exacerbated by the stigma of a suicide attempt. In our study, however, we did not find a statistically significant increase in linkage to MH services after brief interventions. Thus, their protective effects may operate primarily through indirect psychosocial mechanisms rather than through direct increases in treatment uptake. Given that this finding was based on only six studies, these results should be interpreted with caution. Future studies with larger data sets are needed to clarify the role of BICs in promoting MH service engagement.

Yet, several critical questions remain unanswered for advancing suicide prevention research, such as: “Which BICs are most effective for specific outcomes?” and “Who benefits most from which type of intervention?” In addressing the first question, our subgroup analysis suggested some variation in effects across intervention types, although meta-regression analyses did not detect statistically significant differences. Brief interventions were the most consistently associated with reductions in suicide attempts and suicidal ideation, whereas evidence for multimodal or remote contact interventions was sparse or highly heterogeneous, precluding firm conclusions regarding comparative effectiveness. These findings indicate that the limited number of studies and uneven distribution of intervention types likely contributed to the lack of significant effects observed in the meta-regression, rather than true equivalence of interventions. On a descriptive level, we found that the studies (14, 39%) that found the intervention to be effective for the outcomes of suicide deaths,^83^ re-attempts,^40^^,^^66^ suicidal behavior,^35^^,^^72^^,^^79^ self-harm,^55^^,^^62^^,^^66^^,^^78^ and suicidal thoughts^36^^,^^54^^,^^68^^,^^81^^,^^82^^,^^100^ comprised interventions with the following characteristics: CBT-based approaches (with or without medication), problem-solving, psychoeducation, volitional help-sheet, psychodynamic-based approaches, ASSIP, empowerment-focused approach, and remote contact interventions lasting 12 months or more. This aligns with prior findings that identified the following key components: safety planning, psychoeducation, long-term follow-up, care coordination, early engagement,^9^^,^^14^^,^^15^ social support, and suicide prevention literacy.^13^ Notably, CBT-based psychotherapeutic interventions have been repeatedly linked to reductions in suicide re-attempts compared with control conditions.^101^

For the question: “Who benefits most?”, we found no significant effects of population on the outcomes. Similarly, meta-regression analyses showed no significant differences between intervention formats (ultra-brief vs. brief) or by year of publication, indicating that BIC effects may be relatively consistent across different clinical and non-clinical groups and over the two-decade span of included studies. This suggests that BICs’ effects may be relatively consistent across different clinical and non-clinical groups. While this could indicate broad applicability of the interventions, it should be considered that the small sample available for the meta-regressions and the substantial heterogeneity limited the generalizability of the results.

Previous findings are mixed: while some align with our study,^10^^,^^19^^,^^28^ others have found that high-risk groups, including ED patients or psychiatric inpatients, benefit particularly from active outreach.^97^^,^^102^ Overall, heterogeneity across BIC trials, in terms of population, intervention type, dose, and follow-up, limits conclusions about which components are most effective. Although our meta-regression analyses did not reveal significant moderators, likely due to small subgroup sizes, more detailed future analyses could clarify differential effects. The lack of consistent sex- and gender-specific reporting across trials further limited our ability to assess whether intervention effects differed by sex or gender.

These uncertainties are further reflected in the GRADE assessment. Despite all outcomes being considered primary, the certainty of evidence was predominantly rated as low to very low, primarily due to serious imprecision resulting from small sample sizes and wide confidence intervals, as well as additional concerns related to risk of bias and inconsistency. Consequently, confidence in the magnitude of observed effects remains limited, and future well-powered, methodologically robust trials are likely to influence both the certainty and potentially the direction of effect estimates.

Clinically, these findings highlight the use of BICs as scalable, accessible interventions for suicide prevention. Their brevity and low resource demands make them suitable for settings such as emergency departments, primary care, or community outreach, where traditional psychotherapy may not be feasible. Providers can integrate BICs into post-crisis care to quickly reduce suicide re-attempts and address acute suicidal ideation. While specific intervention types may not substantially alter outcomes, tailoring delivery to patient needs and context can enhance engagement. However, given the low certainty of evidence, implementation should be accompanied by careful monitoring and integration with more intensive interventions when indicated. Variability in staff training, organizational contexts, and intervention fidelity may further influence effectiveness, underscoring the importance of implementation research alongside efficacy trials.

This work has several limitations. First, seven studies were rated as high risk of bias, and 22 as some risk. While this warrants caution, sensitivity analyses showed that excluding high-risk studies did not meaningfully alter effect sizes or significance (see Supplementary Table S7). Second, the multivariate meta-analyses examining the effects of the follow-up period showed substantial heterogeneity, suggesting that additional moderators may influence the results, an issue that future research should explore. Third, combining psychotherapeutic interventions with psychosocial interventions might have introduced heterogeneity. Psychotherapeutic and psychosocial BICs differ in theoretical foundations, required qualifications, and likely mechanisms of action. To explore these differences, we conducted subgroup analyses and a meta-regression with intervention type as a moderator, providing initial evidence on potential variation in effects across modalities. Nonetheless, future studies with more detailed intervention classifications are needed to clarify differential impacts. Last, heterogeneity was also present in sample sizes (24–1699 participants), follow-up periods (3 weeks–2 years), control conditions (active vs. TAU), and intervention approaches. Studies were grouped by intervention type to address this, but small numbers in some categories limited power in meta-regression analyses. Further research is needed to clarify the role of intervention type and population.

A major strength of this review is the summary of the available evidence on BICs relevant to clinicians and researchers alike. It allows secondary prevention health care providers to better navigate and assess the available BIC options for patients after a suicide attempt. By clarifying where evidence for efficacy exists and where uncertainties remain, providers are better equipped to make more informed treatment decisions. Additionally, our work contributes to the ongoing debate on intervention effectiveness, showing that BICs were equally effective, independent of intervention type and treated subgroup.

In summary, brief intervention contacts can help reduce suicide re-attempts and suicidal ideation, though effects on acute suicidal thoughts may wane over time. Their low intensity, scalability, and flexibility support integration into emergency and post-discharge care, including delivery by trained non-clinical staff to maintain continuity. Nevertheless, uncertainties remain regarding effect size and underlying mechanisms, and BICs should be considered adjunctive components within broader, system-level suicide prevention strategies. Future well-powered comparative trials and standardised reporting will be essential to optimize intervention design and guide evidence-based clinical implementation.

## Contributors

SH, BK, RCO, PH, and SO conceptualized the study. SH, PH, BK, and RCO were responsible for the methodology. SH, LB, SM, MM, AP, and PH have access to and verify the underlying study data. SH did the formal analysis. MM, SM, AMB, CR, AP, LK, and LB provided the resources. RS provided expert guidance on the methodology and validation of the search string. SH wrote the first draft. All co-authors reviewed and edited the manuscript. BK and ROC provided supervision.

## Data sharing statement

All data used in this study are available from the original publications included in this meta-analysis. No new participant-level data were generated.

## Declaration of interests

Birgit Kleim also received Grants or contracts from the Swiss National Science Foundation project MULTICAST (501100001711-205913). Rory C. O'Connor also reports grants or contracts from the Medical Research Foundation, the Mindstep Foundation, Chief Scientist Office, Medical Research Council, Public Health Scotland, Scottish Government, NIHR. Shout 85258, Scottish Association for Mental Health, Zoetis Foundation, Jonathan's Voice, ADHD UK, and the Barfil Charitable Trust, royalties or licenses from books on suicide, and occasional honoraria for lectures/workshops. Rory C. O'Connor is a Trustee and Science Council Member of MQ Mental Health Research, Chair of the Academic Advisory Group to the Scottish Government's National Suicide Prevention Advisory Group, and a board member of the International Academy of Suicide Research. Rory C. O'Connor was a member of the National Institute for Health and Care Excellence's guideline group for the management of self-harm. Sebastian Olbrich reports Honoraria from Janssen and Schwabe Pharma, Idorsia for lectures, presentations, speakers bureaus, manuscript writing, or educational events, and payment for expert testimony from Janssen outside the submitted work. All other authors declare no competing interests.

## Appendix A Supplementary data

Translated Abstract
