# Supplementary material for: Effectiveness of brief interventions and contacts after suicide attempt: a systematic review and meta-analysis
Source: eClinicalMedicine. 2026 Mar 12;93:103824. doi: 10.1016/j.eclinm.2026.103824 (PMC12996265; doi:10.1016/j.eclinm.2026.103824)
Supplement: Translated Abstract [file mmc1.docx]

*The following translations in German were submitted by the authors and we reproduce them as supplied. They have not been peer reviewed. Our editorial processes have only been applied to the original abstract in English, which should serve as reference for this manuscript*

Zusammenfassung (German translation)

**Hintergrund** Nach einem Suizidversuch erhält nur ein Drittel der Betroffenen eine ambulante Behandlung, was den Bedarf an niedrigschwelligen Kurzinterventionen und Kontakten, im Folgenden als Kurzinterventionen abgekürzt, deutlich macht. Wir wollten die Wirksamkeit dieser Kurzinterventionen untersuchen.

**Methoden** In dieser systematischen Übersicht und Metaanalyse haben wir wissenschaftliche Datenbanken und graue Literatur nach randomisierten kontrollierten Studien zu Kurzinterventionen und Kontakten bei Erwachsenen nach einem Suizidversuch seit Beginn bis zum 18. Juni 2025 durchsucht. Es wurden Studien einbezogen, die die Wirksamkeit von Kurzinterventionen im Vergleich zu einer Kontrollgruppe bei Erwachsenen (im Alter von 18 bis 65 Jahren) nach einem Suizidversuch untersuchten, mit dem Ziel, Wiederholungen von Suizidversuchen, Suizidgedanken, selbstverletzendes Verhalten und nicht-suizidale Selbstverletzungen zu reduzieren oder die Anbindung an psychiatrisch-psychotherapeutische Versorgungsangebote zu erhöhen. Die primären Outcomes umfassten erneute Suizidversuche, selbstverletzendes Verhalten, Suizidgedanken, nicht-suizidale Selbstverletzungen und die Anbindung an psychiatrisch-psychotherapeutische Versorgungsangebote. Wir extrahierten die rohen Häufigkeitszahlen und Mittelwerte/Standardabweichungen für die Verwendung in Meta-Analysen von Odds Ratios bzw. standardisierten Mittelwertdifferenzen. Die Qualität der Evidenz wurde mit RoB 2 und GRADE bewertet. Diese Studie wurde bei PROSPERO unter der Nummer CRD42022271143 registriert.

**Ergebnisse** Es wurden 36 Studien (9552 Teilnehmer; 1993–2025) eingeschlossen; davon waren 33 für die Meta-Analysen geeignet. Die Zahl der erneuten Suizidversuche war nach Kurzinterventionen im Vergleich zur Kontrollgruppe signifikant reduziert (Evidenz mit moderater Sicherheit; OR = 0.72, 95% KI [0.54, 0.95]; I^2^ = 56.8 %; n = 23 Studien). Nach Kurzinterventionen wurde im Vergleich zur Kontrollgruppe eine Reduktion der Suizidgedanken beobachtet (Evidenz mit geringer Sicherheit; SMD = -0.20, 95 % KI [-0.36, -0.05]; I^2^ = 63.4 %; n = 15 Studien). Es fand sich keine Evidenz für eine Reduktion der Wiederholung von selbstverletzendem Verhalten (Evidenz mit sehr geringer Sicherheit; OR = 0.82, 95% KI [0.22, 1.97]; I^2^ = 80.9 %; n = 4 Studien) und einer Zunahme der Anbindung an psychiatrisch-psychotherapeutische Versorgungsangebote (Evidenz mit sehr niedriger Sicherheit; OR = 2.25, 95% KI [0.71, 7.17]; I^2^ = 89.8 %; n = 6 Studien). Für eine Untersuchung von nicht-suizidalen Selbstverletzungen standen zu wenige Studien zur Verfügung. Die meisten Studien hatten jedoch einen moderaten Risk of Bias (22 Studien, 61 %), während einige wenige Studien sogar einen hohen Risk of Bias zeigten (7 Studien, 19 %). Die Faktoren Risk of Bias, Heterogenität und Ungenauigkeit führten insgesamt zu einer Herabstufung der Evidenzsicherheit.

**Interpretation** Unsere Ergebnisse haben wichtige Implikationen für die klinische Praxis und Suizidprävention. Moderate, gesicherte Evidenz stützt die Annahme, dass bereits eine einzige Sitzung mit Kurzinterventionen das Wiederauftreten von Suizidversuchen wirksam reduzieren kann, und weniger gesicherte Evidenz deutet darauf hin, dass sie auch Suizidgedanken verringern können. Obwohl die sehr geringe Evidenzsicherheit Schlussfolgerungen hinsichtlich des Wiederauftretens von selbstverletzendem Verhalten und der Anbindung an psychiatrisch-psychotherapeutische Versorgungsangebote einschränkt, können diese Interventionen als praktischer und potenziell wesentlicher Bestandteil von Suizidpräventionsstrategien angesehen werden. Dennoch sind weitere hochwertige Studien erforderlich, um die Wirkungen auf zusätzliche Ergebnisse und Populationen zu bestätigen.

**Finanzierung** Die Studie wurde gefördert durch Mittel des Schweizerischen Nationalfonds (501100001711-205913), der EMDO-Stiftung der Universität Zürich, des HOLCIM-Unternehmens zur Förderung der wissenschaftlichen Weiterbildung.

**Schlüsselwörter** Kurzinterventionen und Kontakte, psychosoziale Intervention, Suizidversuch, randomisierte kontrollierte Studien, Metaanalyse.
