# Supplementary material for: Effectiveness of brief interventions and contacts after suicide attempt: a systematic review and meta-analysis
Source: eClinicalMedicine. 2026 Mar 12;93:103824. doi: 10.1016/j.eclinm.2026.103824 (PMC12996265; doi:10.1016/j.eclinm.2026.103824)
Supplement: Supplementary Figures and Tables [file mmc2.pdf]

Supplementary material to “**Effectiveness of brief interventions and contacts after suicide attempt: a systematic review and meta-analysis**”

This file contains

- Figures 1-10
- Tables 1-16

Methods

We followed the PRISMA-P guidelines[1] and adhered to the recommendations outlined in the Cochrane Handbook for Systematic Reviews of Interventions[2].

Criteria for study selection

Study types. We included all randomized controlled trials (RCTs) that evaluated specific brief psychosocial interventions compared with a control condition, such as treatment as usual (TAU), routine psychiatric care, enhanced usual care, active comparators, or their combinations. Eligible study designs comprised traditional RCTs, cluster-RCTs, and cross-over trials, regardless of publication type or language. Quasi-randomized trials were excluded.

Participants. We included adult participants (aged 18 and older) of all ethnicities and sexes who sought treatment following a suicide attempt. "Suicide attempt" was defined in line with Silverman and colleagues[3] as a "self-inflicted, potentially injurious behavior with a nonfatal outcome, for which there is evidence (either explicit or implicit) of intent to die." Trials including participants with suicidal ideation only, i.e., without a documented history of suicide attempts, were excluded. Consequently, we also excluded trials that specified the inclusion of participants with "suicidal thoughts and/or after a suicide attempt."

Interventions. Brief interventions were defined as structured, short-term approaches to reduce suicide risk, including 1–4 session ultra-brief or 6–12 session brief formats[4]. Delivered in person or online, they involved psychotherapy or brief contacts (e.g., calls, postcards) to promote engagement, crisis help-seeking, and timely support. Based on the categories from a prior review[5], we grouped interventions as follows:

- Brief interventions: psychotherapy-based approaches, safety planning, and psychoeducation.
- Remote contact interventions: follow-up through letters, green cards, postcards, phone calls, emergency cards, or crisis cards.
- Multimodal interventions: combinations of brief interventions and contact-based elements.
- Other: interventions focused solely on psychoeducation or maintaining contact.

Comparators. Our primary comparator was treatment as usual (TAU), defined according to Witt and colleagues' definition[5] as the standard care a patient would receive outside of a study context. We also included studies that used active comparison interventions alongside TAU.

Data extraction

All relevant data were independently extracted by two researchers. In cases of missing or incomplete information, we contacted the corresponding authors for clarification. SH compared extracted data, and any discrepancies were resolved through discussion.

We collected the following data:

- General information: first author, title, year, design, country, study aim, and key conclusions;
- Participants: population description, suicide attempt method, clinical setting, inclusion criteria, diagnosis, assessment tools, numbers randomized/analyzed, age, and sex distribution;
- Intervention: type, format, number of sessions, and follow-up details (period and frequency);
- Comparator: type, format, session count, and follow-up details;
- Outcomes: primary/secondary outcomes, raw data, and timing of assessments.
- For the primary endpoint, we used the time point specified for the primary outcome. If unspecified, we extracted data from the time point closest to intervention completion, aligning with our focus on post-intervention effectiveness.

Synthesize of evidence

Brief interventions. Brief therapeutic interventions can be broadly categorized into two types: (1) primary interventions and (2) secondary interventions. Primary interventions are designed to directly target STBs, whereas secondary interventions focus on associated psychopathology, aiming to reduce STBs as an indirect consequence. Interventions classified as primary included those centered on forming implementation intentions[16,23], brief CBT-based programs[17,24–28], psychodynamic-based therapies[29,30], and a DBT-informed approach[31]. Other primary strategies involved promoting early engagement with services and strengthening protective factors[32,33], structured safety planning interventions[34], and learning to disclose ones suicide story[35]. Secondary interventions, by contrast, included psychosocial approaches such as problem-solving training[17,36,37] which aimed to address underlying psychological vulnerabilities contributing to suicidal behavior.

Remote contact interventions. Several studies[18,38–47] investigated different forms of brief contacts, which comprised letters, green cards, postcards, telephone calls, emergency cards, and crisis cards. Green cards, used in studies such as[42], provided access to a 24/7 on-call psychiatrist for crisis telephone consultation. Coping cards[47] contained personalized therapeutic strategies for managing distress, promoting easy reference during crises. Crisis cards, used in several studies[39,40,42,43,46], summarized a patient's treatment plan in anticipation of situations where the individual may be unable to recall or articulate this information due to acute illness[48].

Multimodal interventions. All studies[20,21,49,50] in this category examined the same intervention: the Attempted Suicide Short Intervention Program (ASSIP). ASSIP[51] comprises three structured psychotherapy sessions designed to rapidly establish a therapeutic alliance, provide psychoeducation, deliver a cognitive case formulation, and develop an individualized safety plan. This is followed by sustained long-term contact, consisting of 12 personalized letters sent over a two-year period.

Other. The studies investigated psychoeducation combined with ongoing contact maintenance[52,53], culturally adapted psychoeducation[54], and brief admission for intensive therapy based on a problem-solving approach[55].

Date: June 18th, 2025			
PICO	#	Searches	Results
Population	1	Suicide, Attempted/	24534
	2	suicid* adj3 (attempt* OR fail* OR intent* OR behavior* OR behaviour*).ti,ab,kf.	26419
	3	1 or 2	36895
	4	Drug Overdose/ or Opiate Overdose/	16305
	5	(overdose* or over dose*).ti,ab,kf.	31039
	6	4 or 5	35779
Intervention	7	(Psychosocial Intervention.mp. or Crisis Intervention/ or Psychotherapy, Brief/ or Suicide Prevention/ or Psychological First Aid/ or Emergency Services, Psychiatric/ or Secondary Prevention/mt)	32155
	8	((psychosocial OR crisis OR crises OR risk OR brief OR short-term OR suicid* OR emergency OR secondar*) adj2 (interven* OR treat* OR prevent* OR therap* OR psychotherap* OR care* OR service* OR aid*)).ti,ab,kf.	251493
	9	7 or 8	267385
Outcomes	10	Self-Injurious Behavior/ or Self Mutilation/ or Suicidal Ideation/ or Suicide Prevention/ or Poisoning/	61252
	11	(parasuicid* or auto mutilat* or automutilat* or self destruct* or selfdestruct* or self-harm* or selfharm* or self immolat* or selfimmolat* or self inflict* or selfinflict* or self injur* or selfinjur* or selfmutilat* or self mutilat* or self poison* or selfpoison* or (suicid* adj3 attempt*) or (suicid* adj3 re-attempt*) or (suicid* adj2 ideation*) or (suicid* adj2 thought*) or self-harm* or self-injur* or nssi or ((nonsuicid* or non-suicid*) adj2 (self*))).ti,ab,kf.	63969
	12	10 or 11	99655
	13	Patient Acceptance of Health Care/sn or Mental Health Services/ or Community Mental Health Services/ or Counseling/ or Social Work, Psychiatric/	120608
	14	((accept* or linkage or start* or connect* or begin* or util* or usag* or "use" or using or adher* or help seek*) adj2 (mental health service* or treat* or care or therap* or psychotherap* or counsel* or intervent*)).ti,ab,kf.	**440869**
	15	13 or 14	550335
Population	16	3 or 6	70159
outcomes	17	12 or 15	645282
RCT	18	(("adaptive clinical trial" or "clinical trial" or "clinical trial, phase i" or "clinical trial, phase ii" or "clinical trial, phase iii" or "clinical trial, phase iv" or "controlled clinical trial" or "equivalence trial" or "multicenter study" or "pragmatic clinical trial" or "randomized controlled trial").pt. or "adaptive clinical trials as topic"/ or "Clinical Studies as Topic"/ or "clinical trials as topic"/ or "clinical trials, phase i as topic"/ or "clinical trials, phase ii as topic"/ or "clinical trials, phase iii as topic"/ or "clinical trials, phase iv as topic"/ or "controlled clinical trials as topic"/ or "double-blind method"/ or "early termination of clinical trials"/ or "equivalence trials as topic"/ or "intention to treat analysis"/ or "multicenter studies as topic"/ or "non-randomized controlled trials as topic"/ or "pragmatic clinical trials as topic"/ or "randomized controlled trials as topic"/ or (("2" or two or "3" or three or "4" or four) adj arm).ti,ab,kf,kw. or ((clinical or controlled or (cross adj over) or crossover or equivalent or pragmatic or randomisation or randomised or randomization or randomized) adj3 (studies or study or trial or trials)).ti,ab,kf,kw. or (cluster* adj3 (randomised or randomized)).ti,ab,kf,kw. or (stepped adj wedge).ti,ab,kf,kw. or ((window adj opportunity) and trial).ti,ab,kf,kw. or (phase adj1 ("I" or "II" or "III" or "IV" or "1" or "2" or "3" or "4")).ti,ab,kf,kw. or ((one or two or three or four or single or doubl* or tripl*) adj4 (blind or blinded or mask or masked)).ti,ab,kf,kw. or (intervention or interventions).ti. or ((random adj1 assignment*) or (randomly adj1 assigned)).ti,ab,kf,kw.) not ((exp animals/ or exp plants/) not humans/)	2653327
adults	19	(adult not (child or adolescent)).sh.	4121733
	20	9 and 16 and 17 and 18 and 19	323
	21	9 and 16 and 17 and 18	1317
	22	remove duplicates from 21	1316

Supplementary Figure 1. *MEDLINE search history as an example for the search strings.*

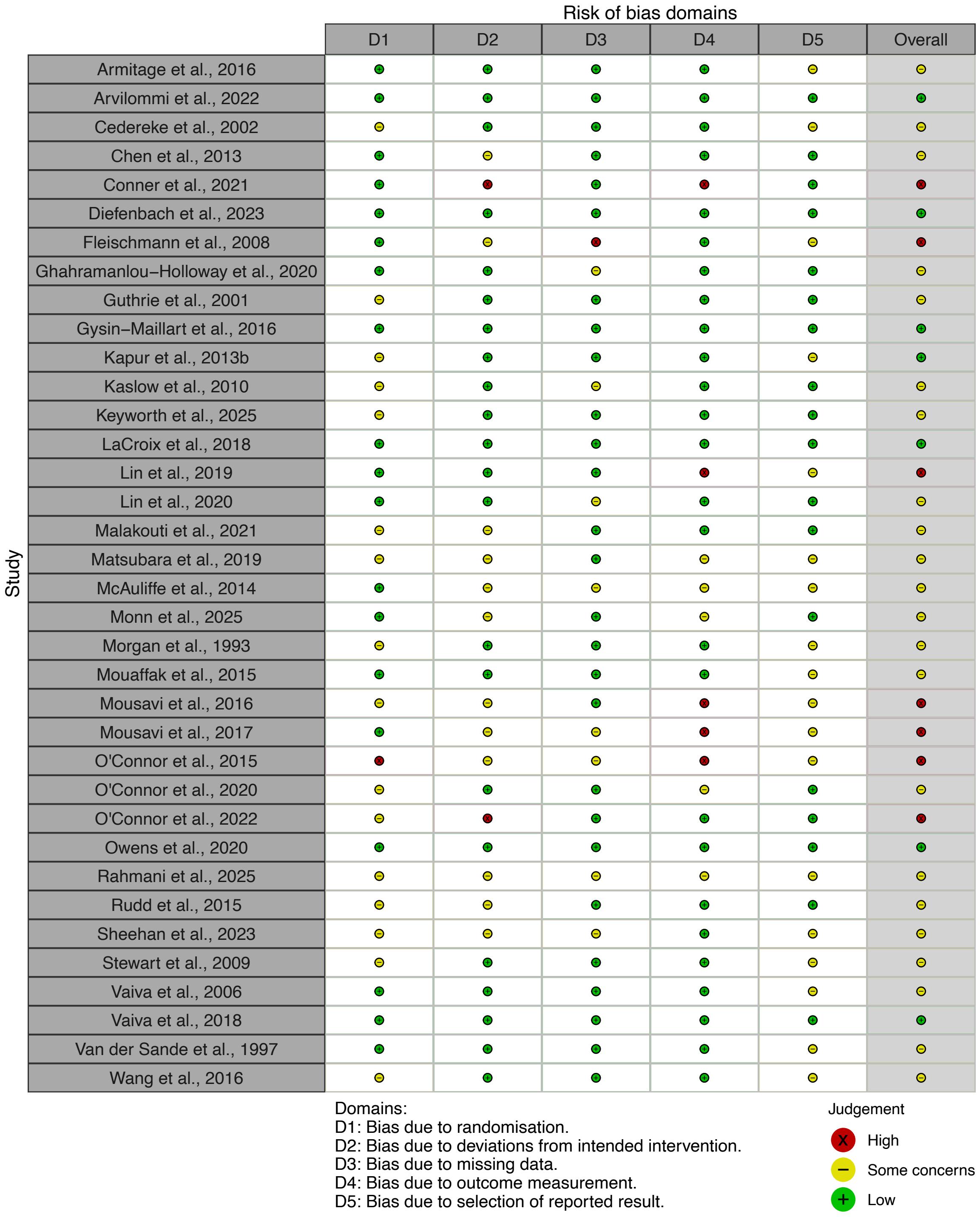

Supplementary Figure 2. ***Overview of Risk of Bias Assessments.*** The bar chart illustrates the results of the risk of bias assessments across all studies, categorized into three groups: 'low risk,' 'high risk,' and 'some concerns.' The chart was generated using the robvis package in R (McGuinness, 2019).

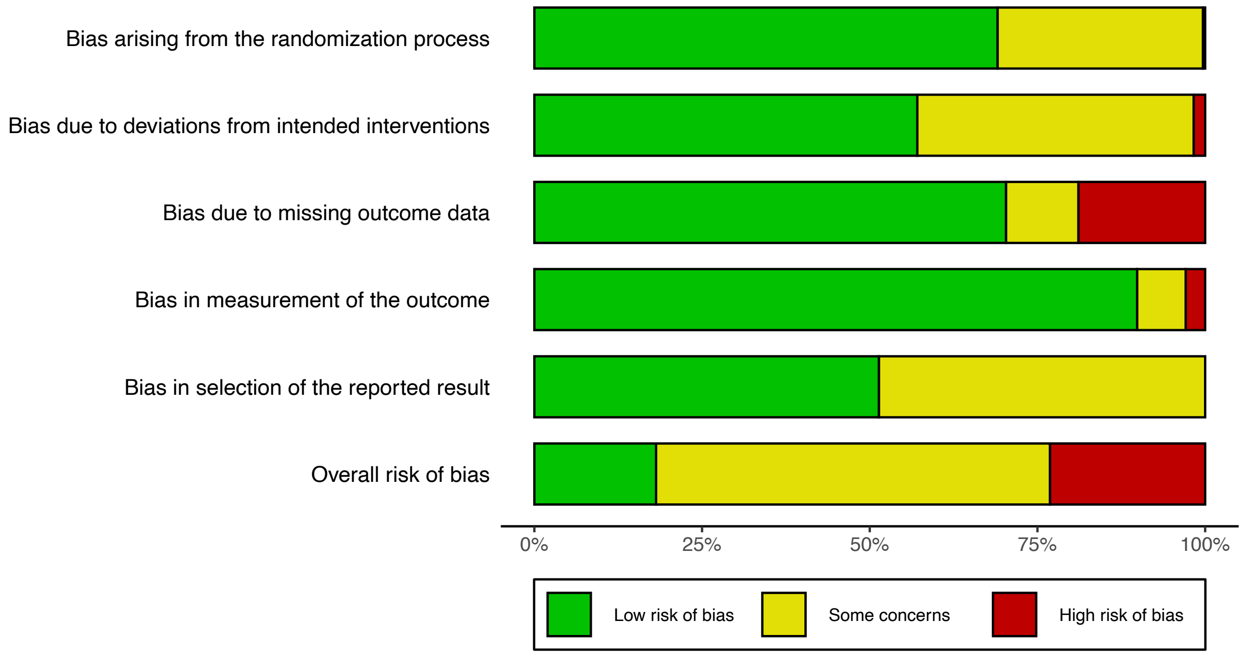

Supplementary Figure 3. ***Risk of bias assessment.*** The traffic light plot shows the results of the 'risk oof bias' assessment for each study.

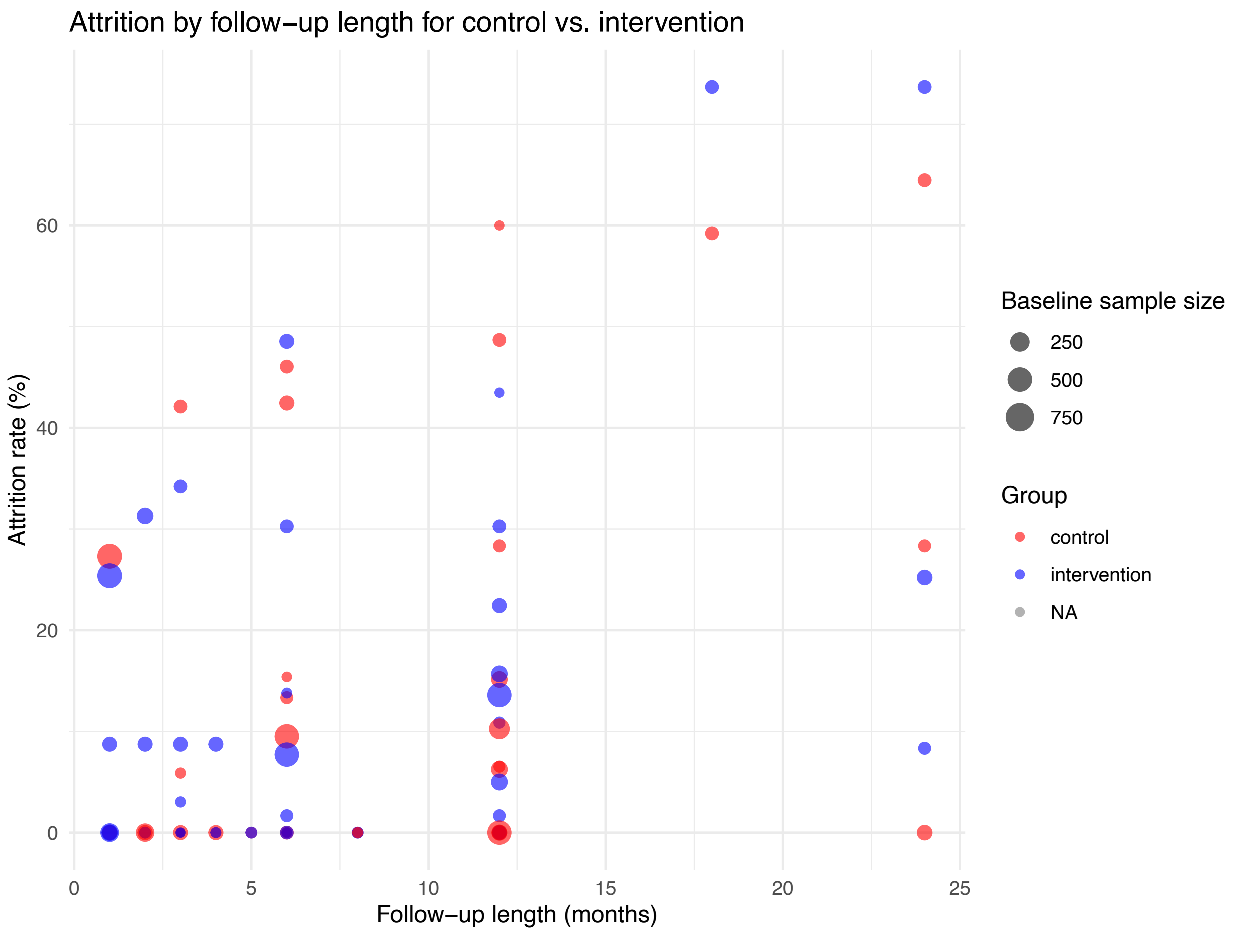

Supplementary Figure 4. ***Attrition against follow-up length.*** Attrition rates across studies by follow-up length. Each point represents a study's attrition at a given follow-up month, with bubble size proportional to the study's baseline sample size. Colors indicate study arm (blue = intervention, red = control). Attrition rates were calculated as the proportion of participants who did not complete the follow-up relative to the baseline sample. Studies with longer follow-up periods tended to show higher attrition.

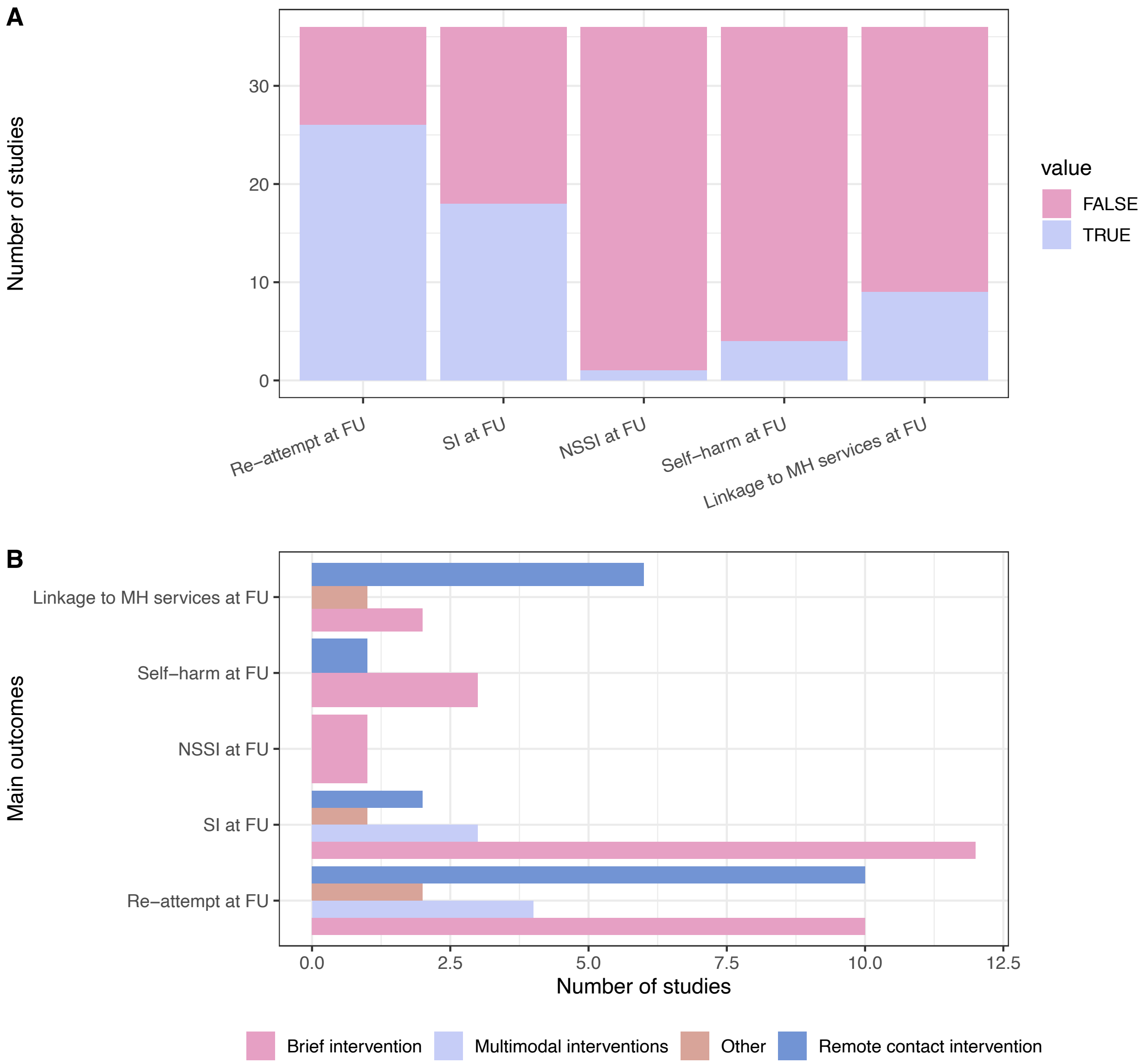

Supplementary Figure 5. ***Descriptive plots.*** Panel (A) shows the distribution of the studies that investigated the outcomes of interest 'suicide re-attempts at follow-up', 'suicidal ideation at follow-up', 'NSSI at follow-up', 'self-harm at follow-up', and 'linkage to mental health service at follow-up'. Panel (B) shows the distribution of the intervention types by outcome.

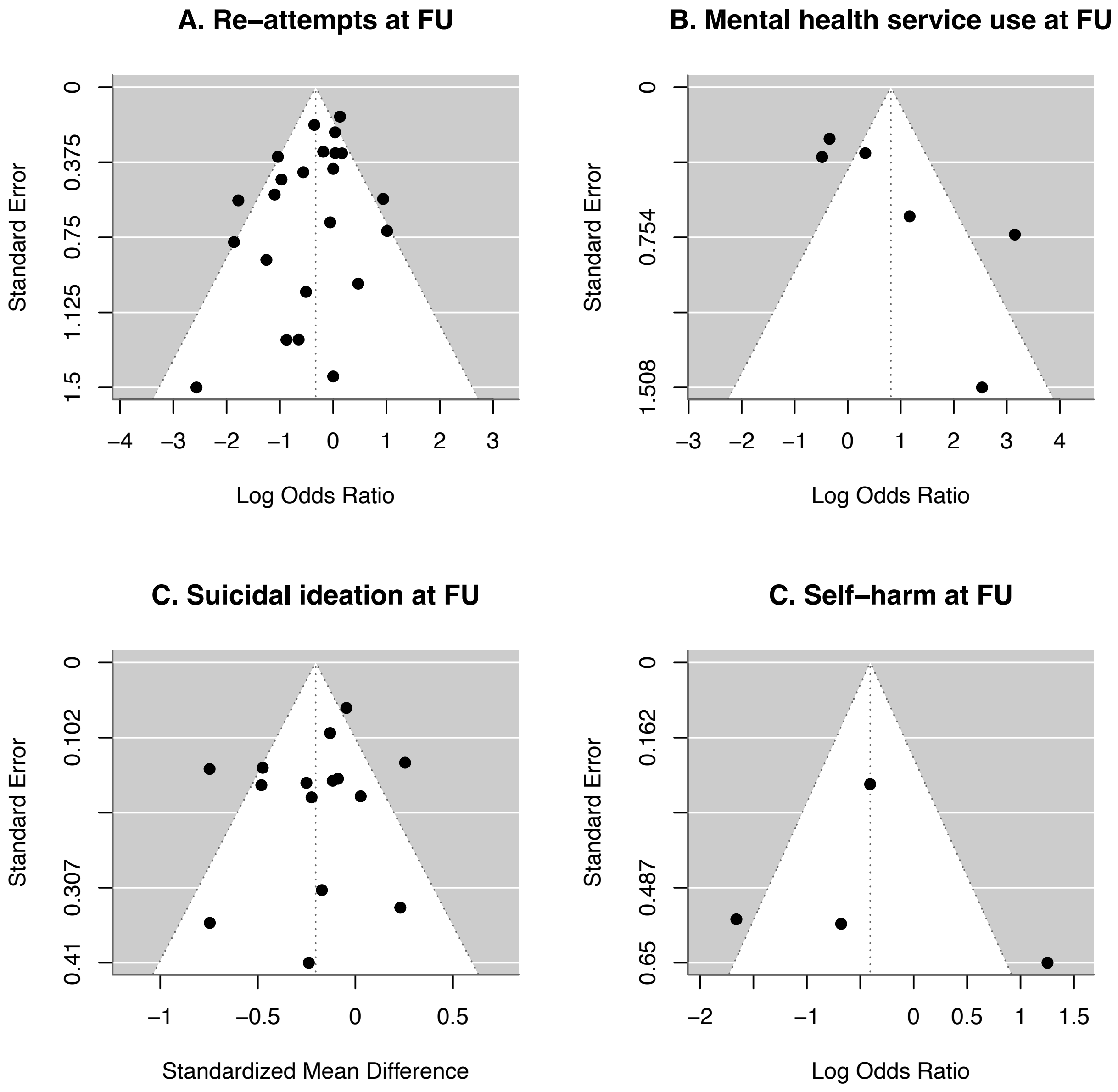

Supplementary Figure 6. ***Funnel plots.*** The funnel plots show the observed effect sizes on the x-axis against some measure of precision of the observed effect sizes, here the standard error, on the y-axis. In the absence of publication bias and heterogeneity, the points representing the individual studies should fall inside the pseudo-confidence region[56]. Funnel plots across all outcomes did not show clear evidence of publication bias. Although panels B and C exhibit mild dispersion or apparent asymmetry, these patterns were small, inconsistent across outcomes, and likely attributable to the limited number of studies and substantial between-study heterogeneity rather than true small-study effects. The Peters/Eggers test indicated no small study effects for any of the outcomes, except 'linkage to MH service at follow-up' ($z = 3.23$, $p < .001$). This suggested that smaller studies tended to report larger effect sizes. Visual inspection of the funnel plots confirmed this asymmetry.

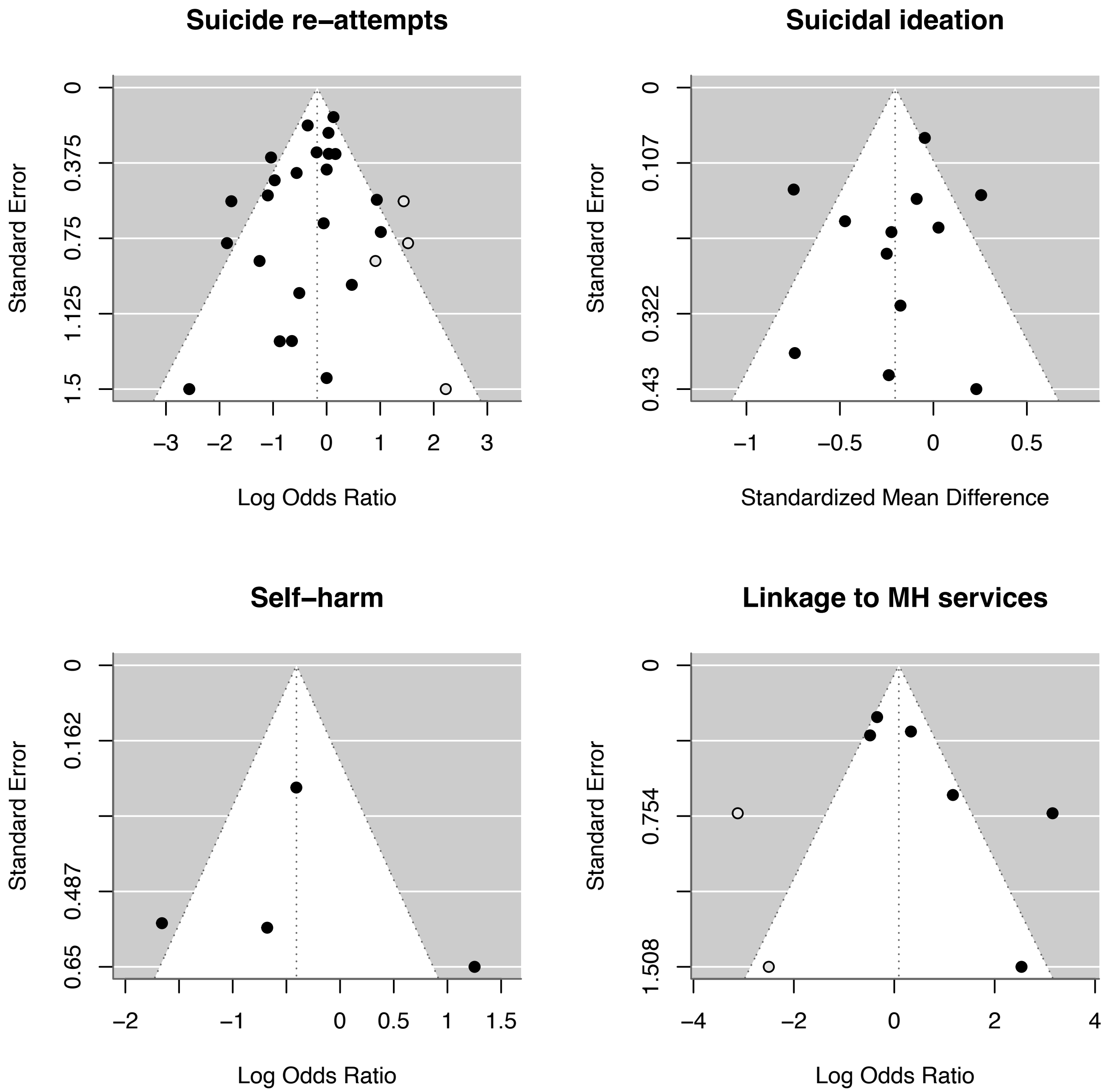

Supplementary Figure 7. ***Trim-and-fill plots for all four outcomes included in the meta-analysis.*** Observed studies are shown as filled circles, and imputed studies, representing potentially missing studies identified by the trim-and-fill analysis, are shown as open circles. The method provides an exploratory assessment of publication bias by estimating the number and location of missing studies needed to restore funnel plot symmetry and recalculating an adjusted pooled effect size. Trim-and-fill results should be interpreted cautiously, particularly in the presence of substantial heterogeneity or a small number of studies.

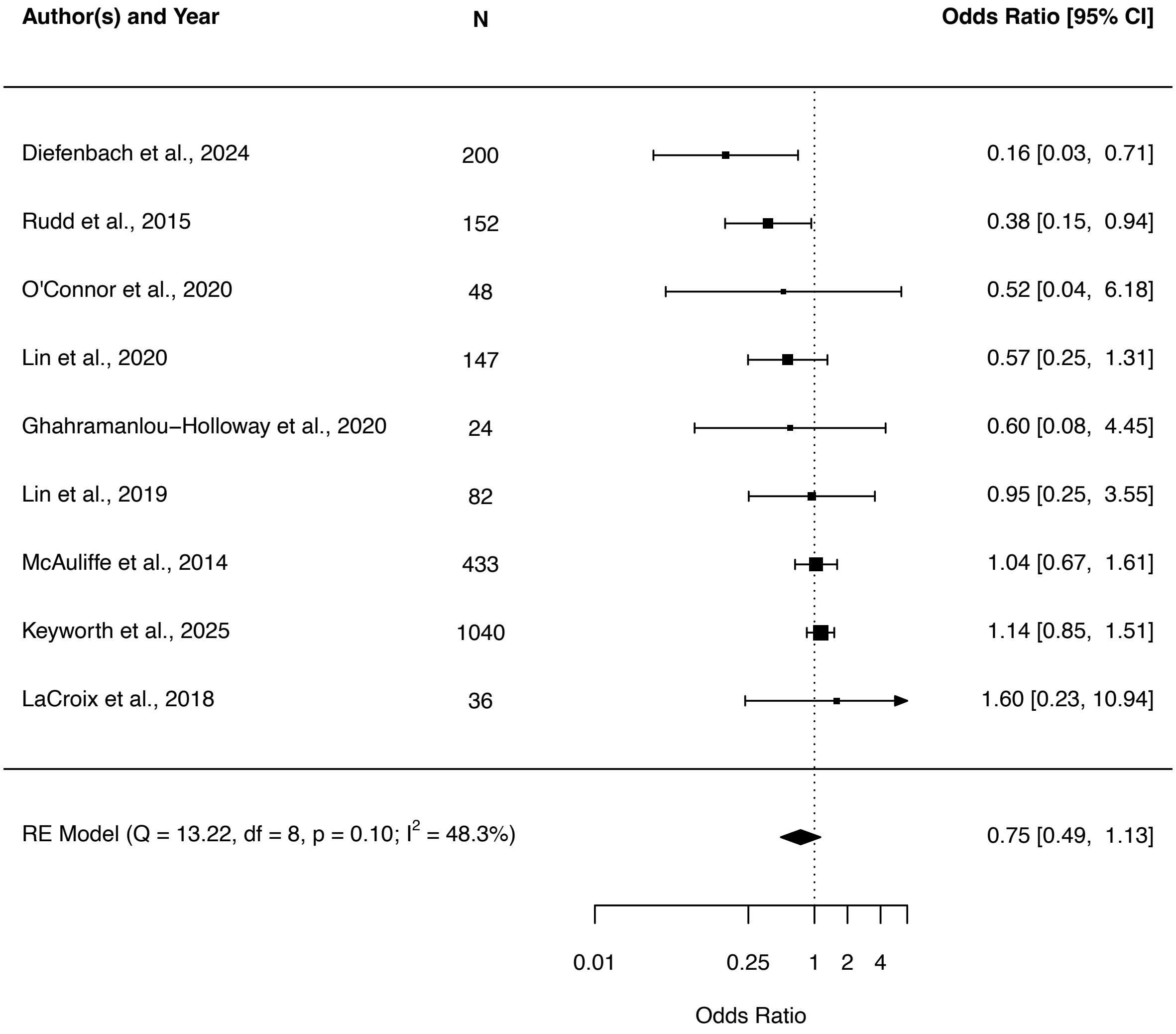

Supplementary Figure 8. ***Suicide attempt: Subgroup analysis by intervention type.*** The meta-analysis for the intervention type 'brief intervention'. The forest plot presents the odds ratios (OR) for the meta-analyses of categorical outcomes along with the corresponding 95% confidence intervals (CIs) for brief interventions and contacts versus control.

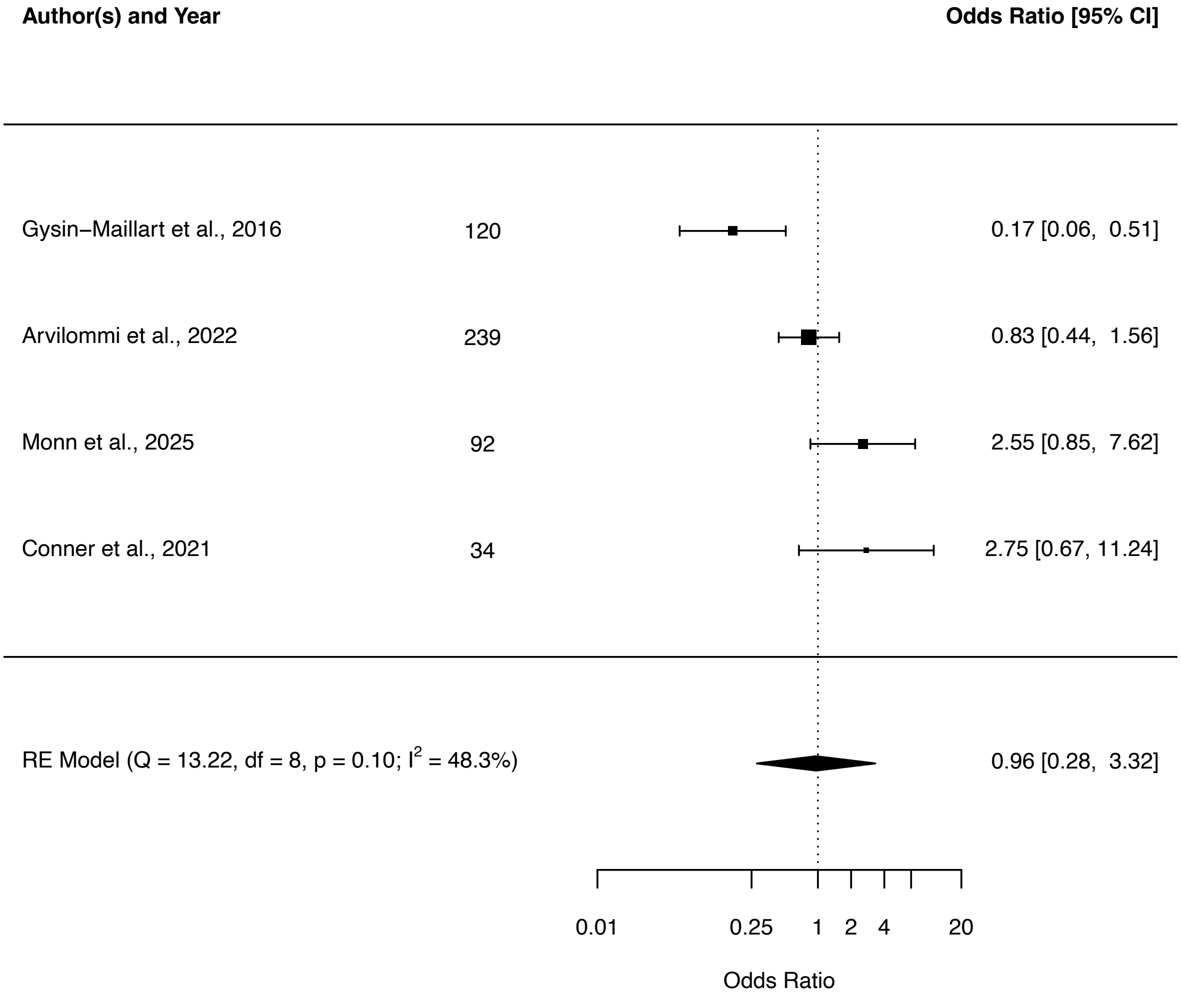

Supplementary Figure 9. ***Suicide attempt: Subgroup analysis by intervention type.*** The meta-analysis for the intervention type 'multimodal intervention'. The forest plot presents the odds ratios (OR) for the meta-analyses of categorical outcomes along with the corresponding 95% confidence intervals (CIs) for brief interventions and contacts versus control.

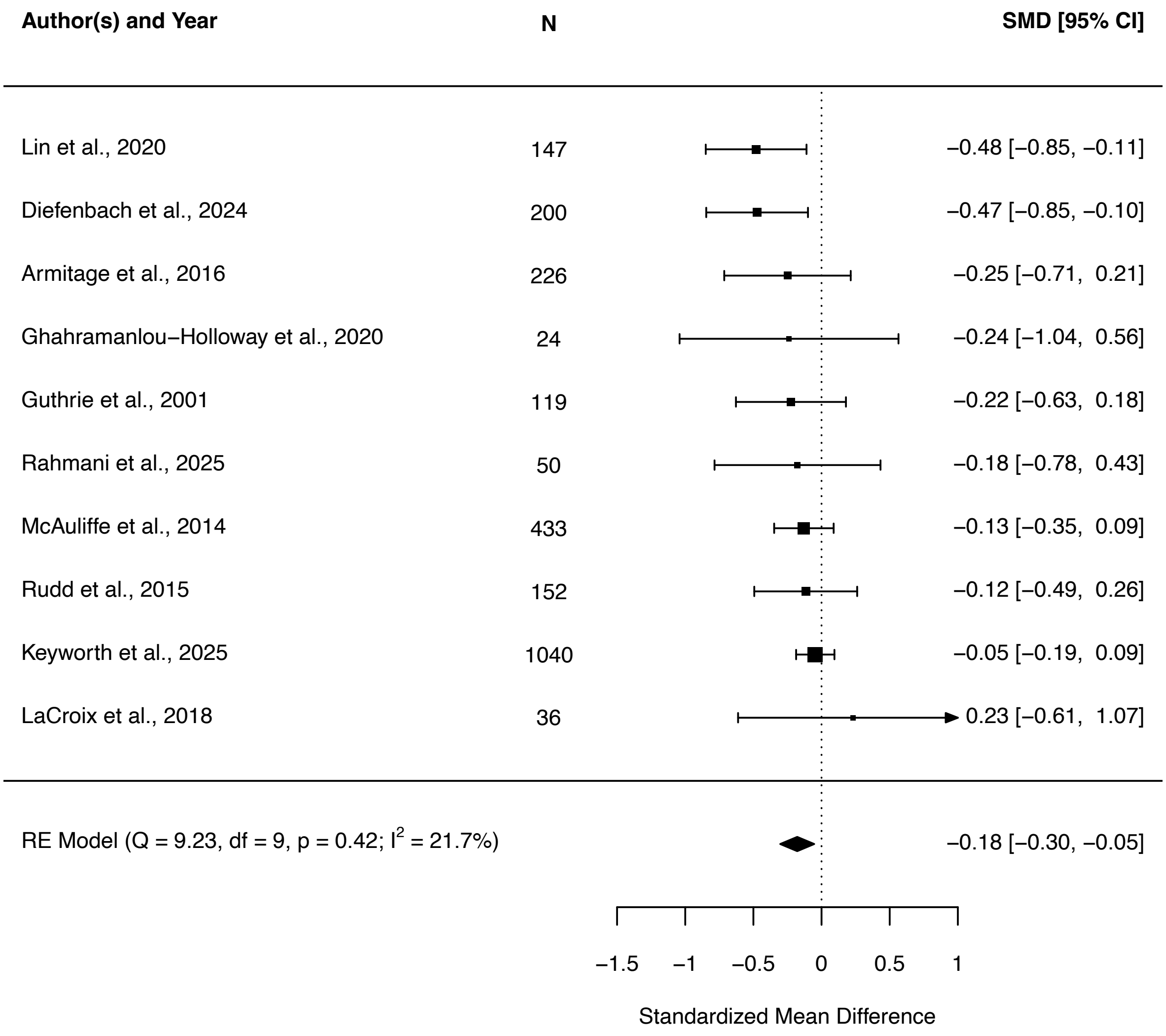

Supplementary Figure 10. ***Suicidal ideation: Subgroup analysis by intervention type.*** The meta-analysis for the intervention type ‘brief intervention’. The forest plot presents the standardized mean differences (SMD) for the meta-analyses of continuous outcomes along with the corresponding 95% confidence intervals (CIs) for brief interventions and contacts versus control.

Supplementary Tables

Supplementary Table 1

Overview of all studies excluded during the title-abstract stage.

Authors (Date)	Title	Reason for Exclusion	Further reasons for exclusion
Armitage et al. (2016)	An exploratory randomised trial of a simple brief psychological intervention to reduce subsequent suicidal ideation and behaviour in patients admitted to hospital for self-harm	wrong population (no suicide attempt)	NA
Arvilommi et al. (2021)	A randomized clinical trial of the ASSIP vs. Crisis counselling in preventing suicide attempt repetition: a two-year follow-up study	wrong study design (conference abstract)	NA
Arvilommi et al. (2022)	ASSIP vs. Crisis counseling for preventing suicide re-attempts: Outcome predictor analysis of a randomized clinical trial data	wrong study design (secondary analysis)	NA
Baker et al. (2024)	Telehealth brief congitive behavioral therapy for suicide prevention: A randomized clinical trial	wrong population (no suicide attempt)	NA
Barnhofer et al. (2015)	Mindfulness-Based Cognitive Therapy (MBCT) reduces the association between depressive symptoms and suicidal cognitions in patients with a history of suicidal depression	wrong population (no suicide attempt)	NA
Bateman et al. (2016)	A randomised controlled trial of mentalization-based treatment versus structured clinical management for patients with comorbig borderline personality disorder and antisocial personality disorder	wrong population (no suicide attempt)	no brief intervention
Bentley et al. (2017)	Treating suicidal thoughts and behaviors within an emotional disorders framework: Acceptability and feasibility of the Unified Protocol in an inpatient setting	wrong population (no suicide attempt)	NA
Bertolote et al. (2010)	Repetition of suicide attempts: Data from emergency care settings in five culturally different low- and middle-income countries participating in the WHO SUPRE-MISS Study	wrong study design (secondary analysis)	NA
Brown et al. (2005)	Cognitive therapy for the prevention of suicide attempts: A randomized controlled trial	wrong population (wrong age)	NA
Bryan et al. (2017)	Effect of crisis response planning vs. Contracts for safety on suicide risk in U.S. army soldiers: A randomized clinical trial	wrong population (no suicide attempt)	NA
Bryan et al. (2018)	Differential effects of brief CBT vs. Treatment as usual on posttreatment suicide attempts among groups of suicidal patients	wrong population (no suicide attempt)	secondary analysis
Bryan et al. (2018)	Effect of crisis response planning on patient mood and clinician decision making: A clinical trial with suicidal U.S. soldiers	wrong study design (secondary analysis)	NA
Bryan et al. (2018)	Mechanisms of action contributing to reductions in suicide attempts following brief cognitive behavioral therapy for military personnel: A test of the interpersonal-psychological theory of suicide	wrong population (no suicide attempt)	NA

Authors (Date)	Title	Reason for Exclusion	Further reasons for exclusion
Cebria et al. (2015)	Telephone management program for patients discharged from an emergency department after a suicide attempt	wrong study design (no randomization)	NA
Celano et al. (2017)	Psychological interventions to reducde suicidality in high-risk patients with major depression: a randomized controlled trial	wrong population (no suicide attempt)	no brief intervention
Chesin et al. (2023)	Using mindfulness-based cognitive therapy to prevent suicide among high suicide-risk patients who also misuse opioids: a preliminary probe of feasibility and effectiveness	wrong study design (secondary analysis)	NA
Comtois et al. (2019)	Effect of augmenting standard care for military personell with brief caring text messages for suicide prevention: a randomized clinical trial	wrong population (no suicide attempt)	NA
Demesmaeker et al. (2019)	Impacts d'un système de veille en prévention du sicide sur les consommations de soins	foreign language	NA
Diefenbach et al. (2025)	The effect of inpatient brief cognitive-behavioral therapy for suicide prevention on post-discharge emergency departement utilization: Secondary analysis of a randomized clinical trial	wrong study design (secondary analysis)	NA
Dimeff et al. (2021)	Using a tablet-based app to deliver evidence-based practices for suicidal patients in the emergency departement: Pilot randomized controlled trial	wrong population (no suicide attempt)	NA
Ducasse et al. (2018)	Gratitude diary for the management of suicidal inpatients: A randomized controlled trial	wrong population (no suicide attempt)	NA
Ducasse et al. (2018)	Acceptance and Commitment Therapy for the management of suicidal patients: a randomized controlled trial	wrong population (no suicide attempt)	NA
Gabilondo et al. (2020)	Prevention of suicidal behavior with telemedicine in patients with a recent suicide attempt: Is a 6-month intervention long enough?	wrong study design (no randomization)	NA
Guthrie et al. (2003)	Predictors of outcome following brief psychodynamic interpersonal therapy for deliberate self-poisoning	wrong study design (secondary analysis)	NA
Hassanian-Moghaddam et al. (2011)	Postcards in Persia: randomised controlled trial to reduce suicidal behaviours 12 months after hospital-treated self-poisoning	wrong population (wrong age)	NA
Hatcher et al. (2011)	Problem-solving therapy for people who present to hospital with self-harm: Zelen randomised controlled trial	wrong population (no suicide attempt)	wrong age
Husain et al. (2023)	A culturally adapted manual-assisted problem-solving intervention (CMAP) for adults with a history of self-harm: a multi-centre randomised controlled trial	wrong population (no suicide attempt)	NA
Ilgen et al. (2022)	Encouraging the use of the Veterans Crisis Linse among high-risk Veterans: A randomized trial of a Crisis Line Facilitation intervention	wrong population (no suicide attempt)	NA

Authors (Date)	Title	Reason for Exclusion	Further reasons for exclusion
Interian et al. (2024)	Mindfulness-Based Cognitive Therapy for preventing suicide in military Veterans: A randomized clinical trial	wrong population (no suicide attempt)	NA
Jobes et al. (2017)	A randomized cotrolled trial of the collaborative assessment and management of suicidality versus enhanced care as usual with suicidal soldiers	wrong population (no suicide attempt)	NA
Jones et al. (2025)	A digital dialectical behaviour therapy intervention for acute suicidality in psychiatric inpatients: A feasibility randomised controlled study	wrong population (no suicide attempt)	NA
Josifovski et al. (2022)	A pilot study of a text message and online brief contact intervention following self-harm or a suicide attempt: A mixed methods evaluation	wrong study design (no randomization)	NA
Lahoz et al. (2016)	Preventing repetition of attempted suicide - III. The Amager Project, 5-year follow-up of randomized controlled trial	wrong population (wrong age)	NA
Lockwood et al. (2025)	Pragmatic randomised controlled trial of two brief community practice-based interventions for self-harm and suicidal ideation	wrong population (no suicide attempt)	NA
Lohani et al. (2024)	Collaboration matters: a randomized controlled trial of patient-clinician collaboration in suicide risk assessment and intervention	wrong population (no suicide attempt)	NA
Luxton et al. (2020)	Caring E-mails for Military and Veteran suicide prevention: A randomized controlled trial	wrong population (no suicide attempt)	NA
MacLeod et al. (1998)	Recovery of positive future thinking within a high-risk parasuicide group: Results from a pilot randomized controlled trial	wrong population (wrong age)	NA
Marasinghe et al. (2012)	Effect of mobile phone-based psychotherapy in suicide prevention: a randomized controlled trial in Sri Lanka	wrong population (wrong age)	NA
Miller et al. (2016)	The coping long term with active suicide programm: Description and pilot data	wrong intervention (no brief intervention)	NA
Milner et al. (2019)	The relationship between and electronic mental health stigma campaign and suicidal thoughts and behaviours: a two-arm randomized controlled trial in the Australian contruction industry	wrong population (no suicide attempt)	NA
Morley et al. (2014)	The efficacy of an opportunistirc cognitive behavioral intervention package (OCB) on substance use and comorbid suicide risk: A multisite randomized controlled trial	wrong population (no suicide attempt)	NA
Mousavi et al. (2013)	The efficacy of telephoni follow up in prevention of suicidal reattempt in patients with suicide attempt history	wrong population (wrong age)	NA
Myers et al. (2024)	Initial evalulation for a personalized advantage index to determine which individuals may benefit from mindfulness-based congitive therapy for suicide prevention	wrong population (no suicide attempt)	NA

Authors (Date)	Title	Reason for Exclusion	Further reasons for exclusion
Nakagawa et al. (2009)	Follow-up study of suicide attempters who were given crisis intervention during hospital stay: Pilot study	wrong study design	NA
Park et al. (2018)	Cost-effectiveness of a Brief Structured Intervention program aimed at preventing repeat suicide attempts among those who previously attempted suicide: A secondary analisis of the ASSIP randomized clinical trial	wrong study design (secondary analysis)	NA
Pashak et al. (2022)	Awareness isn't saving lives: An experimental exploration of suicide risk reduction methods for emerging adults	wrong population (no suicide attempt)	wrong study design
Pfeiffer et al. (2025)	Peer support intervention for suicide prevention among high-risk adults in Michigan: A randomized clinical trial	wrong population (no suicide attempt)	NA
Pistorello et al. (2020)	A randomized controlled trial of the Collaborative Assessment and Management of Suicidality (CAMS) versus treatment as usal (TAU) for suicidal college students	wrong population (no suicide attempt)	NA
Pratt et al. (2015)	Cognitive-behavioural suicide prevention for male prisoners: a pilot randomized controlled trial	wrong population (no suicide attempt)	wong intervention (no brief intervention)
Reger et al. (2024)	Caring letters sent by a clinician or peer to at-risk veterans: a randomized clinical trial	wrong population (no suicide attempt)	NA
Rhee et al. (2005)	Efficacy of brief telephone psychotherapy with callers to a suicide hotline	wrong population (no suicide attempt)	NA
Roberge et al. (2019)	Variables associated with reductions in insomnia severity among acutely suicidal patients receiving brief cognitive behavioral therapy for suicide prevention	wrong population (no suicide attempt)	wrong study design (secondary analysis)
Santel et al. (2023)	The Collaborative Assessment and Management of suicidality compared to enhanced treatment as usual for inpatients who are suicidal: A randomized controlled trial	wrong population (no suicide attempt)	NA
Soni et al. (2025)	Recruitment rates, retention rates, and follow-up completion in a brief intervention and contact trial for suicidal behavior: a feasibility study	wrong population (no suicide attempt)	NA
Stecker et al. (2023)	Efficacy of CBT for treatment seeking (CBT-TS) in untreated veterans and service members at risk for suicidal behavior	wrong population (no suicide attempt)	NA
Taha et al. (2015)	Effects of a culturally informed intervention on abused, suicidal African American women	wrong study design (secondary analysis)	NA
Tyrer et al. (2003)	Randomized controlled trial of brief cognitive therapy versus treatment as usual in recurrent deliberate self-harm: the POPMACT study	wrong population (no suicide attempt)	NA

Authors (Date)	Title	Reason for Exclusion	Further reasons for exclusion
Tyrer et al. (2004)	Differential effects of manual assisted cognitive behavior therapy in the treatment of recurrent deliberate self-harm and personality disturbance: The POPMACT study	wrong population (no suicide attempt)	NA
Vaiva et al. (2011)	ALGOS: the development of a randomized controlled trial testing a case management algorithm designed to reduce suicide risk among suicide attempters	wrong study design	NA
van Spijker et al. (2018)	Effectiveness of a web-based self-help program for suicidal thinking in an Australian community sample: Randomized controlled trial	wrong population (no suicide attempt)	NA
Vijayakumar et al. (2011)	Intervention for suicide attempters: A randomized controlled study	wrong population (wrong age)	NA
Ward-Ciesielski et al. (2017)	Comparing brief interventions for suicidal individuals not engaged in treatment: A randomized clinical trial	wrong population (no suicide attempt)	NA
Wei et al. (2013)	An intervention and follow-up study following a suicide attempt in the emergency departments of four general hospitals in Shenyang, China	wrong population (wrong age)	NA
Welu (1977)	A follow-up program for suicide attempters: Evaluation of effectiveness	wrong population (wrong age)	NA
Wilson et al. (2022)	Feasibility of peer-delivered suicide safety planning in the emergency department: Results form a pilot trial	wrong population (no suicide attempt)	wrong age
Winter et al. (2007)	A controlled trial of personal construct psychotherapy for deliberate self-harm	wrong population (no suicide attempt)	wrong study design
Yang et al. (2024)	Effectiveness of internet-based cognitive behavioral therapy in reducing suicidality among high-risk patients with depression: a multicenter randomized controlled trial in Korea	wrong population (no suicide attempt)	NA
Zou et al. (2017)	Efficacy of psychological pain theory-based cognitive therapy in suicidal patients with major depressive disorder: A pilot study	wrong population (no suicide attempt)	no brief intervention

Supplementary Table 2

Meta-analyses results for all outcomes post-treatment

Outcome	Estimate	Standard Error	Lower CI	Upper CI	Z-value	P-value
Suicide re-attempts	-0.33	0.14	-0.61	-0.61	-2.29	0.02
Suicidal ideation	-0.20	0.08	-0.36	-0.36	-2.61	0.01
Self-harm	-0.41	0.56	-1.50	-1.50	-0.73	0.47
Linkage to MH service	0.81	0.59	-0.34	-0.34	1.38	0.17

Notes. CI, 95% confidence interval.

Supplementary Table 3

Multivariate meta-analysis results

Model	Outcome	Estimate	SE	CI.lb	CI.ub	p-value	$Tau^{2.1}$	$Tau^{2.2}$
Re-attempts	intercept	-0.510	0.247	-0.995	-0.026	0.039	0.352	0.000
	time	0.019	0.019	-0.018	0.056	0.312	0.000	0.000
Suicidal ideation	intercept	-0.047	0.082	-0.208	0.114	0.568	0.004	0.000
	time	-0.037	0.017	-0.070	-0.004	0.027	0.000	0.030
Self-harm	intercept	-0.544	1.110	-2.721	1.632	0.624	0.000	0.000
	time	0.082	0.118	-0.149	0.313	0.488	0.000	0.345

Notes. CI.lb, confidence interval, lower bound; CI.ub, confidence interval, upper bound; SE, standard error; Tau2.1, between-study variance for the first outcome (after accounting for sampling error); Tau2.2, between-study variance for the second outcome.

Supplementary Table 4a

Suicide attempts: Subgroup results.

Intervention	k	OR	CI lower	CI upper	p value	tau^2	I^2
Brief intervention	9	0.748	0.494	1.133	0.171	0.146	48.3
Multimodal intervention	4	0.963	0.279	3.323	0.953	1.297	83.1

Notes. CI lower, confidence interval, lower bound; CI upper, confidence interval, upper bound; k, number of studies; OR, pooled odds ratio; I^2, Percentage of total variability in effect estimates due to heterogeneity; tau^2, estimated between-study variance (amount of heterogeneity) in the random-effects model.

Supplementary Table 4b

Suicidal ideation: Subgroup results.

Intervention	k	OR	CI lower	CI upper	p value	tau^2	I^2
Brief intervention	10	0.837	0.739	0.948	0.005	0.008	21.7

Notes. CI lower, confidence interval, lower bound; CI upper, confidence interval, upper bound; k, number of studies; OR, pooled odds ratio; I^2, Percentage of total variability in effect estimates due to heterogeneity; tau^2, estimated between-study variance (amount of heterogeneity) in the random-effects model.

Supplementary Table 5

Meta-regression results for suicide re-attempts at FU with intervention type as moderator.

Outcome	Estimate	Standard Error	Lower CI	Upper CI	Z-value	P-value
Intercept	-0.35	0.27	-0.87	-0.87	-1.32	0.19
Multimodal interventions	0.27	0.47	-0.64	-0.64	0.58	0.56
Other	-0.08	0.53	-1.12	-1.12	-0.14	0.89
Remote contact intervention	-0.13	0.40	-0.91	-0.91	-0.32	0.75

Notes. CI, confidence interval.

Supplementary Table 6

Meta-regression results for suicidal ideation at FU with intervention type as moderator.

Outcome	Estimate	Standard Error	Lower CI	Upper CI	Z-value	P-value
Intercept	-0.18	0.06	-0.30	-0.30	-2.82	0.00
Multimodal interventions	0.06	0.15	-0.23	-0.23	0.38	0.70
Remote contact intervention	0.43	0.19	0.07	0.07	2.31	0.02

Supplementary Table 7

Meta-regression results of suicide re-attempts with the studied population as a moderator.

Outcome	Estimate	Standard Error	Lower CI	Upper CI	Z-value	P-value
ED Patients	0.27	0.61	-0.93	1.46	0.44	0.66
Inpatients	-0.09	0.61	-1.28	1.10	-0.15	0.88
Soldiers/Veterans	0.16	0.37	-0.57	0.90	0.44	0.66

Notes. CI, confidence interval.

Supplementary Table 8

Meta-regression results of suicidal ideation with the studied population as a moderator.

Outcome	Estimate	Standard Error	Lower CI	Upper CI	Z-value	P-value
Inpatients	0.11	0.27	-0.42	0.63	0.40	0.69
Soldiers/Veterans	-0.15	0.19	-0.53	0.22	-0.81	0.42

Notes. CI, confidence interval.

Supplementary Table 9

Meta-regression results of linkage to MH services with the studied population as a moderator.

Outcome	Estimate	Standard Error	Lower CI	Upper CI	Z-value	P-value
ED Patients	1.69	1.07	-0.4	3.78	1.58	0.11
Inpatients	1.69	1.07	-0.4	3.78	1.58	0.11

Notes. CI, confidence interval.

Supplementary Table 10

Meta-regression results in suicide re-attempts with BIC type as a moderator

Outcome	Estimate	Standard Error	Lower CI	Upper CI	Z-value	P-value
ultra brief BIC	-0.12	0.39	-0.89	0.65	-0.30	0.77
brief BIC	-0.10	0.36	-0.81	0.61	-0.27	0.79

Notes. CI, confidence interval.

Supplementary Table 11

Meta-regression results in suicidal ideation with BIC type as a moderator

Outcome	Estimate	Standard Error	Lower CI	Upper CI	Z-value	P-value
ultra brief BIC	-0.39	0.19	-0.76	-0.01	-2.02	0.04
brief BIC	-0.32	0.19	-0.70	0.07	-1.62	0.10

Notes. CI, confidence interval.

Supplementary Table 12

Meta-regression results in suicide re-attempts with publication year, intervention type, and population as moderators

Outcome	Subcategory	Estimate	Standard Error	Lower CI	Upper CI	Z-value	P-value
Year of publication		0.00	0.03	-0.06	0.05	-0.12	0.91
	multimodal	0.34	0.66	-0.95	1.62	0.51	0.61
Intervention type	Other BIC	-0.24	0.84	-1.90	1.41	-0.28	0.78
	RCI	-0.21	0.63	-1.44	1.01	-0.34	0.73
	ED patients	-0.47	1.11	-2.65	1.71	-0.42	0.67
	General population	-0.22	1.23	-2.63	2.19	-0.18	0.86
Population	Inpatient soldiers	-0.45	1.18	-2.75	1.86	-0.38	0.71
	Inpatients	-0.17	1.15	-2.43	2.09	-0.15	0.88

Notes. BIC, brief interventions and contacts; CI, confidence interval; ED, emergency department; RCI, Remote contact intervention.

Supplementary Table 13

Meta-regression results in suicidal ideation with publication year, intervention type, and population as moderators

Outcome	Subcategory	Estimate	Standard Error	Lower CI	Upper CI	Z-value	P-value
Year of publication		-0.01	0.01	-0.03	0.02	-0.51	0.61
	multimodal	0.14	0.16	-0.17	0.45	0.89	0.37
Intervention type	other	-0.41	0.23	-0.87	0.05	-1.76	0.08
	RCI	0.54	0.31	-0.06	1.14	1.77	0.08
	General population	0.23	0.18	-0.13	0.58	1.24	0.22
Population	inpatient soldiers	0.13	0.19	-0.24	0.49	0.70	0.49
	Inpatients	-0.16	0.18	-0.50	0.19	-0.89	0.37

Notes. BIC, brief interventions and contacts; CI, confidence interval; ED, emergency department; RCI, Remote contact intervention.

Supplementary Table 14

Meta-regression results in suicidal ideation with publication year as a moderator

Outcome	Estimate	Standard Error	Lower CI	Upper CI	Z-value	P-value
Year of publication	0.07	0.09	-0.11	0.25	0.73	0.46

Supplementary Table 15

Sensitivity analyses without high-risk studies.

Outcome	Estimate	Standard Error	Lower CI	Upper CI	Z-value	P-value
Suicide re-attempts	-0.34	0.15	-0.65	-0.65	-2.21	0.03
Suicidal ideation	-0.16	0.08	-0.31	-0.31	-2.03	0.04

Notes. CI, confidence interval.

Supplementary Table 16

GRADE profiles table.

Author(s): Stephanie Homan, Marta Marciniak, and Leonie Biele

Question: Should BICs vs. control be used for patients after suicide attempt?

Setting: Emergency departments, inpatient psychiatric units, and outpatient follow-up care for adults after a suicide attempt

Bibliography:

- **Suicide re-attempt:** Arvilommi et al., 2022 ; Cedereke et al., 2002 ; Chen et al., 2013 ; Conner et al., 2021 ; Diefenbach et al., 2024 ; Ghahramanlou-Holloway et al., 2020 ; Gysin-Maillart et al., 2016; Keyworth et al., 2025 ; LaCroix et al., 2018 ; Lin et al., 2019, 2020 ; Malakouti et al., 2021 ; Matsubara et al., 2019 ; McAuliffe et al., 2014 ; Monn et al., 2025 ; Morgan et al., 1993 ; Mouaffak et al., 2015 ; Mousavi et al., 2016, 2017 ; O'Connor et al., 2020 ; Rudd et al., 2015 ; Stewart et al., 2009 ; Vaiva et al., 2006, 2018 ; Wang et al., 2016 ; van der Sande et al., 1997
- **Suicidal ideation:** Armitage et al., 2016; Diefenbach et al., 2024; Guthrie et al., 2001 ; Kaslow et al., 2010 ; Keyworth et al., 2025 ; Mousavi et al., 2017 ; O'Connor et al., 2015 ; Rahmani et al., 2025 ; Stewart et al., 2009 ; Arvilommi et al., 2022 ; Cedereke et al., 2002 ; Conner et al., 2021 ; Ghahramanlou-Holloway et al., 2020 ; Gysin-Maillart et al., 2016 ; LaCroix et al., 2018 ; Lin et al., 2020 ; McAuliffe et al., 2014 ; Rudd et al., 2015
- **Self-harm:** Kapur et al., 2013 ; McAuliffe et al., 2014 ; Owens et al., 2020 ; Guthrie et al., 2001
- **Non-suicidal self-injury:** Keyworth et al., 2025
- **Linkage to MH services:** Cedereke et al., 2002 ; Kapur et al., 2013 ; Morgan et al., 1993 ; O'Connor et al., 2020 ; Lin et al., 2020 ; Malakouti et al., 2021 ; Matsubara et al., 2019 ; Mouaffak et al., 2015 ; Vaiva et al., 2006

Certainty assessment							№ of patients		Effect		Certainty	Importance
№ of studies	Study design	Risk of bias	Inconsistency	Indirectness	Imprecision	Other considerations	BICs	control	Relative (95% CI)	Absolute (95% CI)		
**Suicide re-attempt (assessed with: number of SA)**												
23	randomised trials	not serious	serious[a]	not serious	not serious	none	448/2345 (19.1%)	512/2309 (22.2%)	**OR 0.72** (0.54 to 0.95)	**5 fewer per 100** (from 9 fewer to 1 fewer)	⊕⊕⊕◯ Moderate[a]	CRITICAL
**Suicidal ideation (assessed with: severity of SI)**												
15	randomised trials	not serious	serious[a]	not serious	not serious	none	1269	1204	-	SMD **0.2 SD lower** (0.36 lower to 0.05 lower)	⊕⊕⊕◯ Moderate[a]	IMPORTANT

Certainty assessment							№ of patients		Effect		Certainty	Importance
№ of studies	Study design	Risk of bias	Inconsistency	Indirectness	Imprecision	Other considerations	BICs	control	Relative (95% CI)	Absolute (95% CI)		
**Self-harm (assessed with: frequency of SH episodes)**												
4	randomised trials	not serious	very serious[b]	not serious	very serious[c]	none	53/341 (15.5%)	73/325 (22.5%)	**OR 0.67** (0.22 to 1.98)	**6 fewer per 100** (from 16 fewer to 14 more)	⊕◯◯◯ Very low[b,c]	IMPORTANT
**Non-suicidal self-injury**												
1	randomised trials	not serious	not serious	not serious	very serious[d]	none	Non-suicidal self-injury (NSSI) was defined as engagement in deliberate self-injurious behaviors without suicidal intent. The outcome was operationalized as any occurrence of NSSI versus no occurrence during the assessment period, based on self-report or interview data.				⊕⊕◯◯ Low[d]	IMPORTANT
**Linkage to mental health services (assessed with: number of participants linked)**												
6	randomised trials	not serious	very serious[b]	not serious	very serious[c]	publication bias strongly suspected[e]	202/584 (34.6%)	266/772 (34.5%)	**OR 2.26** (0.71 to 7.16)	**20 more per 100** (from 7 fewer to 45 more)	⊕◯◯◯ Very low[b,c,e]	IMPORTANT

CI: confidence interval; **OR:** odds ratio; **SMD:** standardised mean difference

Explanations

a. Visual inconsistency and statistical analysis also showed heterogeneity.

b. Visual inspection of the forest plot and statistical measures indicated considerable heterogeneity, with very high inconsistency across studies ($I^2 > 80\%$), suggesting substantial variability in effect estimates that could not be explained.

c. The pooled effect estimate was based on only a few studies and had a wide confidence interval crossing the line of no effect and including both clinically important benefit and possible harm. The total sample size did not meet the optimal information size, indicating very serious imprecision.

d. The evidence was based on a single study assessed narratively. The absence of a pooled effect estimate and failure to meet optimal information size result in very serious imprecision.

e. Funnel plot asymmetry was detected using regression testing (Egger test $p = 0.001$), suggesting the presence of small-study effects. The direction of asymmetry indicates that smaller studies may overestimate the effect, raising concerns about possible publication bias. The certainty of evidence was downgraded by one level for publication bias.

References

1. Page, M. J. *et al.* The PRISMA 2020 statement: An updated guideline for reporting systematic reviews. *International Journal of Surgery* **88**, 105906 (2021).

2. *Cochrane handbook for systematic reviews of interventions version 6.5 (updated august 2024)*. (Cochrane, 2024).

3. Silverman, M. M., Berman, A. L., Sanddal, N. D., O'carroll, P. W. & Joiner Jr, T. E. Rebuilding the tower of babel: A revised nomenclature for the study of suicide and suicidal behaviors. Part 2: Suicide-related ideations, communications, and behaviors. *Suicide and Life-Threatening Behavior* **37**, 264–277 (2007).

4. Stanley, B., Brodsky, B. & Monahan, M. Brief and ultra-brief suicide-specific interventions. *Focus* **21**, 129–136 (2023).

5. Witt, K. G. *et al.* Psychosocial interventions for self-harm in adults. *Cochrane Database of Systematic Reviews* (2021).

6. Doupnik, S. K. *et al.* Association of suicide prevention interventions with subsequent suicide attempts, linkage to follow-up care, and depression symptoms for acute care settings: A systematic review and meta-analysis. *JAMA Psychiatry* **77**, 1021–1030 (2020).

7. Clark, J. M. *et al.* Improving the translation of search strategies using the polyglot search translator: A randomized controlled trial. *Journal of the Medical Library Association: JMLA* **108**, 195 (2020).

8. Milner, A. J., Carter, G., Pirkis, J., Robinson, J. & Spittal, M. J. Letters, green cards, telephone calls and postcards: Systematic and meta-analytic review of brief contact interventions for reducing self-harm, suicide attempts and suicide. *The British Journal of Psychiatry* **206**, 184–190 (2015).

9. Azizi, H. *et al.* Prevention of re-attempt suicide through brief contact interventions: A systematic review, meta-analysis, and meta-regression of randomized controlled trials. *Journal of Prevention* **44**, 777–794 (2023).

10. Inagaki, M., Kawashima, Y., Yonemoto, N. & Yamada, M. Active contact and follow-up interventions to prevent repeat suicide attempts during high-risk periods among patients admitted to emergency departments for suicidal behavior: A systematic review and meta-analysis. *BMC Psychiatry* **19**, 1–11 (2019).

11. Noh, D., Park, Y.-S. & Oh, E. G. Effectiveness of telephone-delivered interventions following suicide attempts: A systematic review. *Archives of Psychiatric Nursing* **30**, 114–119 (2016).

12. Nuij, C. *et al.* Safety planning-type interventions for suicide prevention: Meta-analysis. *The British Journal of Psychiatry* **219**, 419–426 (2021).

13. Ouzzani, M., Hammady, H., Fedorowicz, Z. & Elmagarmid, A. Rayyan—a web and mobile app for systematic reviews. *Systematic Reviews* **5**, 210 (2016).

14. Sterne, J. A. *et al.* RoB 2: A revised tool for assessing risk of bias in randomised trials. *BMJ* **366**, (2019).

15. Deeks, J. J., Higgins, J. P., Altman, D. G. & Group, C. S. M. Analysing data and undertaking meta-analyses. *Cochrane Handbook for Systematic Reviews of Interventions,* 241–284 (2019).

16. Armitage, C. J., Rahim, W. A., Rowe, R. & O'Connor, R. C. An exploratory randomised trial of a simple, brief psychological intervention to reduce subsequent suicidal ideation and behaviour in patients admitted to hospital for self-harm. *The British Journal of Psychiatry* **208**, 470–476 (2016).

17. Stewart, C. D., Quinn, A., Plever, S. & Emmerson, B. Comparing cognitive behavior therapy, problem solving therapy, and treatment as usual in a high risk population. *Suicide and Life-Threatening Behavior* **39**, 538–547 (2009).

18. Vaiva, G. *et al.* Effect of telephone contact on further suicide attempts in patients discharged from an emergency department: Randomised controlled study. *BMJ* **332**, 1241–1245 (2006).

19. Higgins, T., Julian PT & VA, W. Cochrane handbook for systematic reviews of interventions version 6.4 (updated august 2023). (2023).

20. Arvilommi, P. *et al.* A randomized clinical trial of attempted suicide short intervention program versus crisis counseling in preventing repeat suicide attempts: A two-year follow-up study. *Psychotherapy and Psychosomatics* **91**, 190–199 (2022).

21. Gysin-Maillart, A., Schwab, S., Soravia, L., Megert, M. & Michel, K. A novel brief therapy for patients who attempt suicide: A 24-months follow-up randomized controlled study of the attempted suicide short intervention program (ASSIP). *PLoS Medicine* **13**, e1001968 (2016).

22. Zelen, M. A new design for randomized clinical trials. *New England Journal of Medicine* **300**, 1242–1245 (1979).

23. Keyworth, C., Leather, J. Z., Quinlivan, L., O'Connor, R. C. & Armitage, C. J. Randomised controlled trial of a brief theory-based online intervention to reduce self-harm. *BJPsych Open* **11**, e63 (2025).

24. Diefenbach, G. J. *et al.* Brief cognitive behavioral therapy for suicidal inpatients: A randomized clinical trial. *JAMA Psychiatry* (2024).

25. Ghahramanlou-Holloway, M. *et al.* Inpatient psychiatric care following a suicide-related hospitalization: A pilot trial of post-admission cognitive therapy in a military medical center. *General Hospital Psychiatry* **63**, 46–53 (2020).

26. Lin, Y.-C. *et al.* Brief cognitive-based psychosocial intervention and case management for suicide attempters discharged from the emergency department in taipei, taiwan: A randomized controlled study. *Suicide and Life-Threatening Behavior* **50**, 688–705 (2020).

27. LaCroix, J. M. *et al.* Pilot trial of post-admission cognitive therapy: Inpatient program for suicide prevention. *Psychological Services* **15**, 279 (2018).

28. Rudd, M. D. *et al.* Brief cognitive-behavioral therapy effects on post-treatment suicide attempts in a military sample: Results of a randomized clinical trial with 2-year follow-up. *American Journal of Psychiatry* **172**, 441–449 (2015).

29. Guthrie, E. *et al.* Randomised controlled trial of brief psychological intervention after deliberate self poisoningCommentary: Another kind of talk that works? *BMJ* **323**, 135 (2001).

30. Rahmani, F., Kakaie, N. & Khazaie, H. Effectiveness of intensive short-term dynamic psychotherapy for suicidal ideation and anger in suicidal behavior. *American Journal of Psychotherapy* appi–psychotherapy (2025).

31. Lin, T.-J. *et al.* The effectiveness of dialectical behavior therapy skills training group vs. Cognitive therapy group on reducing depression and suicide attempts for borderline personality disorder in taiwan. *Archives of Suicide Research* **23**, 82–99 (2019).

32. O'Connor, S. S. *et al.* The development and implementation of a brief intervention for medically admitted suicide attempt survivors. *General Hospital Psychiatry* **37**, 427–433 (2015).

33. O'Connor, S. S. *et al.* Pilot randomized clinical trial of the teachable moment brief intervention for hospitalized suicide attempt survivors. *General Hospital Psychiatry* **63**, 111–118 (2020).

34. O'Connor, R. C. *et al.* SAFETEL: A pilot randomised controlled trial to assess the feasibility and acceptability of a safety planning and telephone follow-up intervention to reduce suicidal behaviour. *Pilot and Feasibility Studies* **8**, 156 (2022).

35. Sheehan, L. *et al.* To share or not to share? Evaluation of a strategic disclosure program for suicide attempt survivors. *Death Studies* **47**, 392–399 (2023).

36. McAuliffe, C. *et al.* Group problem-solving skills training for self-harm: Randomised controlled trial. *The British Journal of Psychiatry* **204**, 383–390 (2014).

37. Owens, D. *et al.* Problem-solving therapy rather than treatment as usual for adults after self-harm: A pragmatic, feasibility, randomised controlled trial (the MIDSHIPS trial). *Pilot and Feasibility Studies* **6**, 119 (2020).

38. Cedereke, M., Monti, K. & Öjehagen, A. Telephone contact with patients in the year after a suicide attempt: Does it affect treatment attendance and outcome? A randomised controlled study. *European Psychiatry* **17**, 82–91 (2002).

39. Chen, W.-J. *et al.* Employing crisis postcards with case management in kaohsiung, taiwan: 6-month outcomes of a randomised controlled trial for suicide attempters. *BMC Psychiatry* **13**, 1–7 (2013).

40. Kapur, N. *et al.* Messages from manchester: Pilot randomised controlled trial following self-harm. *The British Journal of Psychiatry* **203**, 73–74 (2013).

41. Matsubara, T. *et al.* Combining phone and postcard brief contact interventions for preventing suicide reattempts: A quasi-randomized controlled trial. *Psychiatry Research* **279**, 395–396 (2019).

42. Morgan, H., Jones, E. & Owen, J. H. Secondary prevention of non-fatal deliberate self-harm: The green card study. *The British Journal of Psychiatry* **163**, 111–112 (1993).

43. Mouaffak, F., Marchand, A., Castaigne, E., Arnoux, A. & Hardy, P. OSTA program: A french follow up intervention program for suicide prevention. *Psychiatry Research* **230**, 913–918 (2015).

44. Mousavi, S. G., Amini, M., Mahaki, B. & Bagherian-Sararoudi, R. Effect of phone call versus face-to-face follow-up on recurrent suicide attempts prevention in individuals with a history of multiple suicide attempts. *Advanced Biomedical Research* **5**, 184 (2016).

45. Mousavi, S. G., Tehrani, M. N. & Maracy, M. The effect of active treatment and visit compared to conventional treatment, on preventing recurrent suicidal attempts: A randomized controlled clinical trial. *Advanced Biomedical Research* **6**, 38 (2017).

46. Vaiva, G. *et al.* Combining postcards, crisis cards, and telephone contact into a decision-making algorithm to reduce suicide reattempt: A randomized clinical trial of a personalized brief contact intervention. *The Journal of Clinical Psychiatry* **79**, 2132 (2018).

47. Wang, Y.-C. *et al.* Coping card usage can further reduce suicide reattempt in suicide attempter case management within 3-month intervention. *Suicide and Life-Threatening Behavior* **46**, 106–120 (2016).

48. Sutherby, K. *et al.* A study of 'crisis cards' in a community psychiatric service. *Acta Psychiatrica Scandinavica* **100**, 56–61 (1999).

49. Conner, K. R. *et al.* Pilot RCT of the attempted suicide short intervention program (ASSIP) adapted for rapid delivery during hospitalization to adult suicide attempt patients with substance use problems. *General Hospital Psychiatry* **72**, 66–72 (2021).

50. Monn, A. *et al.* Randomized controlled trial for the attempted suicide short intervention program (ASSIP): An independent non-replication study. *Journal of Affective Disorders* **382**, 59–67 (2025).

51. Gysin-Maillart, A. *ASSIP – kurztherapie nach suizidversuch: Attempted suicide short intervention program. therapiemanual*. 120 (Hogrefe, 2021). doi:10.1024/86149-000.

52. Fleischmann, A. *et al.* Effectiveness of brief intervention and contact for suicide attempters: A randomized controlled trial in five countries. *Bulletin of the World Health Organization* **86**, 703–709 (2008).

53. Malakouti, S. K. *et al.* Aftercare and suicide reattempt prevention in tehran, iran. *Crisis* (2021).

54. Kaslow, N. J. *et al.* Suicidal, abused african american women's response to a culturally informed intervention. *Journal of Consulting and Clinical Psychology* **78**, 449 (2010).

55. Van Der Sande, R. *et al.* Intensive in-patient and community intervention versus routine care after attempted suicide: A randomised controlled intervention study. *British Journal of Psychiatry* **171**, 35–41 (1997).

56. Sterne, J. A. & Egger, M. Funnel plots for detecting bias in meta-analysis: Guidelines on choice of axis. *Journal of Clinical Epidemiology* **54**, 1046–1055 (2001).
